# F18-Choline PET/CT or MIBI SPECT/CT in the Surgical Management of Primary Hyperparathyroidism: A Diagnostic Randomized Clinical Trial

**DOI:** 10.1001/jamaoto.2024.1421

**Published:** 2024-06-20

**Authors:** Elske Quak, Audrey Lasne-Cardon, Marie Cavarec, Barbara Lireux, Vianney Bastit, Nathalie Roudaut, Pierre-Yves Salaun, Nathalie Keromnes, Gaël Potard, Patricia Vaduva, Annabelle Esvant, Franck Jegoux, Olivier de Crouy-Chanel, Anne Devillers, Clémence Guery, Charline Lasnon, Renaud Ciappuccini, Bérénice Legrand, Adrien Estienne, François Christy, Jean-Michel Grellard, Stéphane Bardet, Bénédicte Clarisse

**Affiliations:** 1 Department of Nuclear Medicine and Thyroid Unit, Centre François Baclesse, Caen, France; 2 Department of Head & Neck Surgery, Centre François Baclesse, Caen, France; 3 Department of Head & Neck Surgery, University Hospital of Caen, Caen France; 4 Department of Nuclear Medicine, University Hospital of Brest, Brest, France; 5 Department of Endocrinology, University Hospital of Brest, Brest, France; 6 EA 3878, University of Bretagne Occidentale, Brest, France; 7 Department of Head & Neck Surgery, University Hospital of Brest, France; 8 Department of Endocrinology, University Hospital of Rennes, Rennes, France; 9 ENT Department, Rennes University Medical Center, Rennes, France; 10 Department of Nuclear Medicine, Eugene Marquis Cancer Institute, Rennes, France; 11 Clinical Research Department, Centre François Baclesse, Caen, France

## Abstract

**Importance:**

Whether F18-choline (FCH) positron emission tomographic (PET)/computed tomographic (CT) scan can replace Tc99m-sestaMIBI (MIBI) single-photon emission (SPE)CT/CT as a first-line imaging technique for preoperative localization of parathyroid adenomas (PTA) in patients with primary hyperparathyroidism (PHPT) is unclear.

**Objective:**

To compare first-line FCH PET/CT vs MIBI SPECT/CT for optimal care in patients with PHPT needing parathyroidectomy and to compare the proportions of patients in whom the first-line imaging method resulted in successful minimally invasive parathyroidectomy (MIP) and normalization of calcemia 1 month after surgery.

**Design, Setting, and Participants:**

A French multicenter randomized open diagnostic intervention phase 3 trial was conducted. Patients were enrolled from November 2019 to May 2022 and participated up to 6 months after surgery. The study included adults with PHPT and an indication for surgical treatment. Patients with previous parathyroid surgery or multiple endocrine neoplasia type 1 (MEN1) were ineligible.

**Interventions:**

Patients were assigned in a 1:1 ratio to receive first-line FCH PET/CT (FCH1) or MIBI SPECT/CT (MIBI1). In the event of negative or inconclusive first-line imaging, they received second-line FCH PET/CT (FCH2) after MIBI1 or MIBI SPECT/CT (MIBI2) after FCH1. All patients underwent surgery under general anesthesia within 12 weeks following the last imaging. Clinical and biologic (serum calcemia and parathyroid hormone levels) assessments were performed 1 and 6 months after surgery.

**Main Outcomes and Measures:**

The primary outcome was a true-positive first-line imaging-guided MIP combined with uncorrected serum calcium levels of 2.55 mmol/l or less 1 month after surgery, corresponding to the local upper limit of normality.

**Results:**

Overall, 57 patients received FCH1 (n = 29) or MIBI1 (n = 28). The mean (SD) age of patients was 62.8 (12.5) years with 15 male (26%) and 42 female (74%) patients. Baseline patient characteristics were similar between groups. Normocalcemia at 1 month after positive first-line imaging-guided MIP was observed in 23 of 27 patients (85%) in the FCH1 group and 14 of 25 patients (56%) in the MIBI1 group. Sensitivity was 82% (95% CI, 62%-93%) and 63% (95% CI, 42%-80%) for FCH1 and MIBI1, respectively. Follow-up at 6 months with biochemical measures was available in 43 patients, confirming that all patients with normocalcemia at 1 month after surgery still had it at 6 months. No adverse events related to imaging and 4 adverse events related to surgery were reported.

**Conclusions:**

This randomized clinical trial found that first-line FCH PET/CT is a suitable and safe replacement for MIBI SPECT/CT. FCH PET/CT leads more patients with PHPT to correct imaging-guided MIP and normocalcemia than MIBI SPECT/CT thanks to its superior sensitivity.

**Trial Registration:**

ClinicalTrials.gov Identifier: NCT04040946

## Key Points

**Question:**

Can F18-choline (FCH) positron emission tomographic (PET)/computed tomographic (CT) scan replace Tc99m-sestaMIBI (MIBI) single-photon emission (SPE)CT/CT as a first-line imaging technique for preoperative localization of parathyroid adenomas (PTA) in patients with primary hyperparathyroidism (PHPT)?

**Findings:**

In this diagnostic randomized clinical trial of 57 assessable patients, normocalcemia 1 month after positive first-line imaging-guided minimally invasive parathyroidectomy (MIP) was more frequent after first-line FCH PET/CT imaging than after first-line MIBI SPECT/CT imaging.

**Meaning:**

These findings suggest that first-line FCH PET/CT can replace MIBI SPECT/CT for imaging-guided surgery in patients with PHPT.

## Teaser

This randomized clinical trial compares first-line F18-choline (FCH) positron emission tomographic (PET)/computed tomographic (CT) scan vs multiplex ion beam imaging (MIBI) single-photon emission CT (SPECT)/CT for optimal care in patients with primary hyperparathyroidism needing parathyroidectomy.

## Introduction

Medical imaging has progressively led to a shortening and simplification of the surgical procedure in primary hyperthyroidism (PHPT). Correct localization of the parathyroid adenoma (PTA) by cross-sectional imaging allows for ambulatory minimally invasive parathyroidectomy (MIP) instead of in-house bilateral cervical exploration (BCE), performed in the event of negative or inconclusive imaging results. MIP is associated with superior cure and lower complication rates than BCE.^1^ Secondary advantages of MIP are shorter operative time, shorter length of stay, lower hospital costs, and favorable cosmetic outcomes.^2^

F18-choline (FCH) PET/CT is a promising new 3-dimensional (3D) nuclear medicine technique for the preoperative localization of parathyroid adenoma (PTA) in patients with PHPT. Its superior detection rate and diagnostic performance compared to the standard 3D technique Tc99m-MIBI (MIBI) SPECT/CT have been suggested in several phase 2 clinical trials and a recent network meta-analysis.^3,4,5,6,7,8,9^ The advantages of FCH PET over MIBI SPECT/CT imaging are the rapid biokinetics of the tracer with a shorter study protocol, superior spatial resolution allowing the detection of small adenomas, and lower exposure of patients to ionizing radiation.^10,11^

The role of FCH PET in the surgical management of PHPT at patient presentation remains to be determined. Currently, FCH PET/CT is not the first-line imaging technique in the preoperative workup of patients with PHPT for the following reasons: FCH has no market authorization for parathyroid imaging, FCH PET/CT is costlier than MIBI SPECT/CT, and FCH PET/CT is generally less available. Furthermore, although FCH PET/CT shows superior diagnostic accuracy over MIBI SPECT/CT, the downstream clinical impact of this difference remains to be investigated.

The APACH2 trial aimed to compare first-line FCH PET/CT vs MIBI SPECT/CT for optimal care in patients with PHPT needing parathyroidectomy. The main objective was to establish the optimal first-line imaging modality resulting in successful MIP and normalization of calcemia.

## Methods

### Study Design and Participants

From November 2019 to May 2022, we conducted a multicenter 2-arm diagnostic intervention randomized phase 3 trial at 4 hospitals in Normandy and Brittany, France. Patients were eligible if they were 18 years or older, had a PHPT diagnosis confirmed by laboratory tests, and an indication for surgical treatment. Exclusion criteria included previous history of parathyroid surgery, a personal or familial history of multiple endocrine neoplasia type 1 (MEN1), pregnancy, breastfeeding, and known allergy for MIBI or FCH or 1 of its excipients.

The trial was approved by the Local Medical Ethics Committee South-East II in July 2019 and by the French Agency for Medical and Health Products Safety in May 2019. The trial protocol has been previously published^12^ (Supplement 1) and was performed in accordance with the ethical standards of the Declaration of Helsinki. All patients gave written informed consent before study entry and any study procedures. The study has been reported in line with the Consolidated Standards of Reporting Trials (CONSORT) reporting guidelines.^13^

### Randomization and Masking

Endocrinologists, nuclear medicine physicians, and surgeons involved in the trial recruited patients who presented with biochemically confirmed PHPT and an indication for surgery. Patients were assigned in a 1:1 ratio to receive either first-line FCH PET/CT (experimental arm) or first-line MIBI SPECT/CT (standard arm). A web-based system was used for electronic randomization and data collection.

### Baseline Evaluations

All eligible patients with signed consent underwent a clinical examination (assessing relevant previous medical history, PHPT history, and current concomitant treatments) and a standard neck ultrasonography before computer-generated randomization. Laboratory tests before randomization included serum calcium, parathyroid hormone (PTH), albumin, phosphorus, 25 hydroxy-vitamin D, creatinine, and creatinine clearance according to the MDRD formula. Pregnancy test was performed before imaging in women of childbearing age.

### Imaging Procedures

Patients randomized to the experimental arm underwent FCH PET/CT (FCH1) of the neck and upper chest 60 minutes after intravenous administration of 1.5 MBq/kg of the radiotracer FCH (details of the imaging procedures are provided in Supplement 2). Patients randomized to the standard arm underwent MIBI SPECT/CT (MIBI1) of the neck and upper chest according to the dual-phase protocol. In the event of negative or inconclusive first-line imaging, crossover imaging was performed: patients with negative/inconclusive FCH1 underwent second-line MIBI SPECT/CT (MIBI2) and patients with negative/inconclusive MIBI1 underwent second-line FCH PET/CT (FCH2). Because FCH is a registered radiotracer only for patients with prostate cancer, any adverse events related to FCH administration in women were recorded.

Two experienced local nuclear medicine physicians (E.Q., M.C., P.S., N.K., A.D., C.G., C.L., R.C., S.B.) interpreted each FCH PET/CT or MIBI SPECT/CT examination in a blinded fashion on Syngo.via workstations (Siemens Healthineers). The result was considered positive in the event of clear focal uptake(s) in a predisposing area. The exact location of each focus was noted (the side and upper or lower position, or the ectopic position) and the maximum transverse CT diameter when measurable. A negative result was defined as the absence of focal uptake. An inconclusive result was defined as faint uptake compared to the surrounding background without CT substrate or uptake most likely related to a thyroid nodule. A concordant result between the 2 raters was directly communicated to the surgeon. In the event of discordance, a third reading was performed at the local interdisciplinary parathyroid meeting and the result was communicated to the surgeon.

### Surgery

All patients underwent surgery under general anesthesia within 12 weeks following the last imaging by an experienced surgeon. In the event of positive imaging results, an outpatient MIP for focused parathyroidectomy was performed. The surgical procedure was adapted in the event of suspected multiple PTAs or ectopic PTA. A conventional inpatient BCE was performed in the event of negative or inconclusive first- and second-line imaging results while first focusing on the more suspected side. All surgical procedures were performed under inferior laryngeal nerve monitoring. The exact location of each resected specimen was noted, as were surgical complications, if any. Vocal cord function was tested by laryngoscopy before discharge.

### Histology

During surgery, an intraoperative frozen section was taken to confirm the presence of parathyroid tissue. Final analysis was performed on paraffin wax–embedded sections stained with hematoxylin and eosin. When necessary, immunohistochemistry with anti-PTH antibody was performed. PTA and parathyroid hyperplasia found at a positive imaging site were considered true positive. PTA and parathyroid hyperplasia found at a negative imaging site were considered false negative. Lesions other than PTA and parathyroid hyperplasia found at a positive imaging site were considered false positive.

### Follow-Up

Clinical and biological (serum values of calcium and PTH) assessments were performed 1 and 6 months after surgery.

### Outcomes

The primary outcome was a true-positive first-line imaging-guided MIP combined with uncorrected serum calcium levels of 10.2 mg/dL (2.55 mmol/L) or less 1 month after surgery, corresponding to the local upper limit of normality.

### Sample Size

The primary end point was the proportion of patients for whom the first-line imaging technique guided the surgical procedure appropriately, ie, toward positive MIP resulting in normocalcemia 1 month after surgery. Based on the results of our previous published phase 2 APACH1 study^5^ in which a sensitivity of around 90% was observed with FCH PET/CT, we opted for a 1-sided test considered to be sufficient in the APACH2 design to demonstrate the superiority of FCH PET/CT over MIBI SPECT/CT, which has a sensitivity of around 60%.^14^ Considering this estimated 30% difference in sensitivity, we calculated that a sample size of 50 evaluable patients (25 per group) would achieve a risk of 5% and a power of 80%. Adjusting for a 15% rate of possible nonassessable patients, we planned to enroll 58 patients overall.

### Statistical Analysis

Clinical and biologic variables were described using the mean, the standard deviation (SD), the minimum and the maximum for continuous data and with percentages for categorical data. Clinical and biologic characteristics of patients were compared between the 2 arms by the χ^2^ test or Fisher exact test for qualitative variables and by the Wilcoxon-Mann-Whitney test for quantitative variables. Calcemia and PTH serum levels were measured at each time point (baseline, 1 month, and 6 months after surgery) and the evolution of these measures were analyzed using the Friedman Rank Sum test. The diagnostic performance of each imaging procedure was assessed by calculating sensitivity and positive predictive value. Interrater reliability for the reading of each imaging was measured using Cohen κ coefficient.^15^

Except for the primary outcome (1-tailed test), all statistical tests were 2-tailed, and *P* values less than .05 were considered statistically significant. Statistical analyses were performed using R statistical software (version 4.1.2; R Foundation) in December 2023.

## Results

### Participants

**Figure 1.**
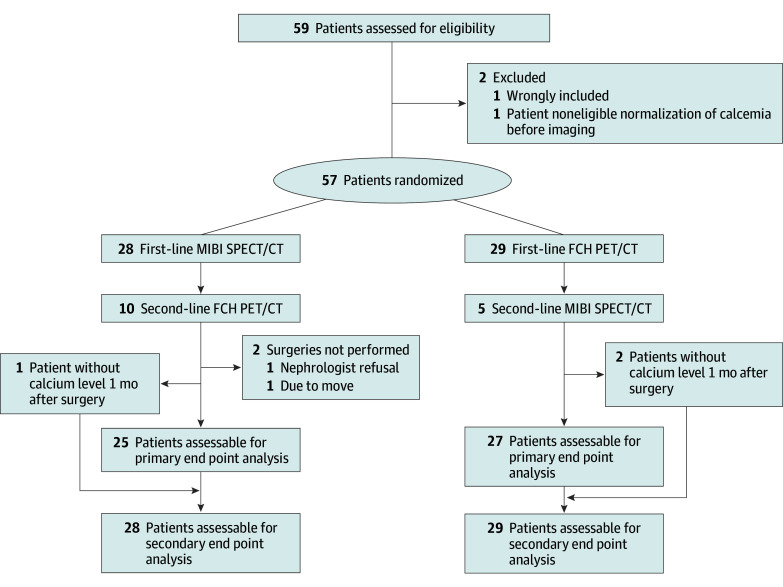
CONSORT Flowchart of Patients Through the Study FCH1 indicates first-line F18-choline (FCH) positron emission tomographic (PET)/computed tomographic (CT) scan PET/CT; MIBI1, first-line Tc99m-sestaMIBI (MIBI) single-photon emission (SPE)CT/CTMIBI SPECT/CT, FCH PET/CT.

Overall, 59 eligible patients provided their informed consent (Figure 1). One patient was not randomized because he was wrongly included and another underwent randomization but did not undergo first-line imaging because of normalized blood tests after randomization. Thus, 57 patients underwent first-line imaging: 29 patients were assigned to FCH1 and 28 patients to MIBI1. Baseline patient characteristics were similar in both groups (Table). Randomization was symmetrical and balanced in all participating centers.

**Table.** Patient Characteristics at Randomization Before First-Line Imaging

Baseline characteristic	FCH1 (N = 29)	MIBI1 (N = 28)	*P *value
Demographic characteristics			
Age, median (range), y	67 (22-85)	64.5 (35-79)	.74
Sex, No. (%)			
Female	21 (72)	21 (75)	.87
Male	8 (28)	7 (25)	
Clinical characteristics			
BMI, mean (SD)	27.4 (6.41)	26.3 (4.05)	.88
BMI missing, No. (%)	7 (24.1)	8 (28.6)	
Calcimimetic treatment			
No. (%)	2 (9)	3 (14)	.66
Missing, No. (%)	6 (20.7)	6 (21.4)	
Previous neck surgery, No. (%)	2 (7)	0	.49
Biological characteristics, mean (SD)			
Calcium, mg/dL	11.08 (0.64)	11.40 (0.96)	.29
PTH, ng/L	136 (56.3)	196 (195)	.29
Phosphorus, ng/L	0.27 (0.05)	0.26 (0.07)	.81
Albumin, g/dL	4.17 (0.37)	4.21 (0.40)	.54
Creatinine, ng/dL	0.79 (0.19)	0.84 (0.28)	.53
Vitamin D, ng/mL	29.9 (11)	24.8 (11.1)	.18

Abbreviations: BMI, body mass index (calculated as weight in kilograms divided by height in meters squared); FCH1, first-line F18-choline (FCH) positron emission tomographic (PET)/computed tomographic (CT) scan PET/CT; MIBI1, first-line Tc99m-sestaMIBI (MIBI) single-photon emission (SPE)CT/CT; PTH, parathyroid hormone. International System of Units (SI) conversion factors for biological measures: calcium, 1 mg/dL = 0.25 mmol/L; PTH, 1 ng/L = 1 pg/mL; phosphorus, 1 ng/L = 3.23 mmol/dL; albumin, 1 g/dL = 10 g/L; creatinine, 1 ng/dL = 88.4 µmol/L.

First-line imaging was positive in 24 patients in the FCH1 group and 18 patients in the MIBI1 group. Five patients randomly assigned to FCH1 had negative or inconclusive results and underwent MIBI2. Ten patients randomly assigned to MIBI1 had negative or inconclusive results and underwent FCH2. All 29 patients in the FCH1 group underwent parathyroid surgery. In the MIBI1 group, 26 of 28 patients underwent parathyroid surgery, 1 did not undergo surgery because of nephrologist refusal and another moved from the region. Follow-up at 1 month was missing for 2 patients in the FCH1 group and for 1 in the MIBI1 group. Follow-up at 6 months was missing for 7 patients in the FCH1 group and 5 in the MIBI1 group (eFigure in Supplement 2).

### Primary Outcome

**Figure 2.**
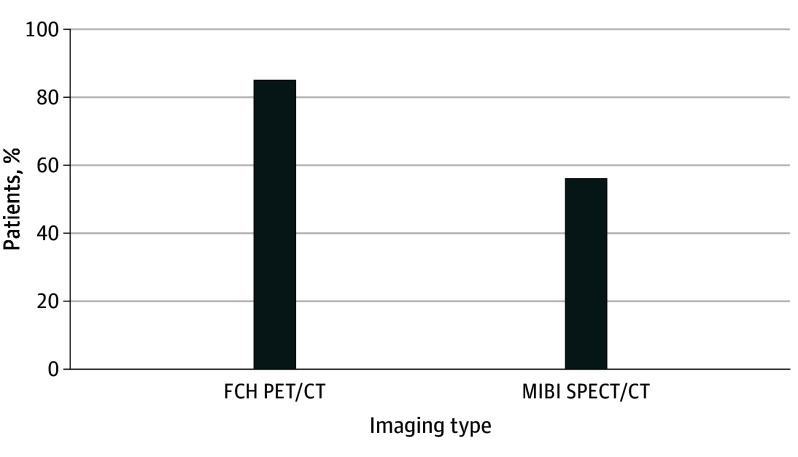
Proportion of Patients With Successful First-Line Imaging-Guided Mini-Invasive Parathyroidectomy and Normocalcemia at 1 Month After Surgery (Main End Point of the Study) FCH PET/CT indicates F18-choline positron emission tomographic/computed tomographic scan; SPECT, single-photon emission CT.

**Figure 3.**
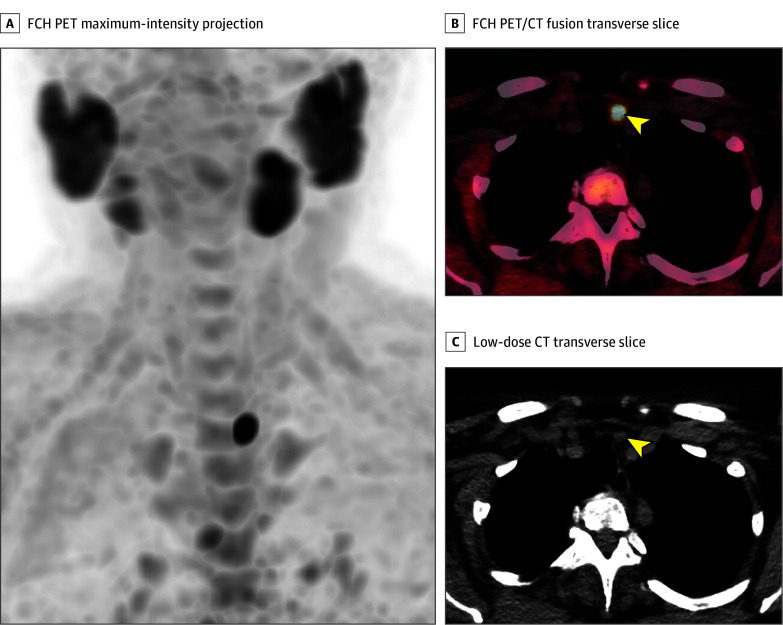
First-Line F18-Choline (FCH) Positron Emission Tomography (PET)/Computed Tomography (CT) Images of a Patient With Primary Hyperparathyroidism (PHPT) A, FCH PET maximum-intensity projection; B, FCH PET/CT fusion transverse slice; and C, low-dose CT transverse slice, showing high FCH uptake in an 11-mm left inferior PTA (arrowheads). The patient underwent minimally invasive parathyroidectomy, leading to normocalcemia during follow-up. No complications occurred.

The primary end point was assessable in 27 patients in the FCH1 group and 25 patients in the MIBI1 group (Figure 2). Calcium levels were normal 1 month after positive first-line imaging-guided MIP in 23 of 27 patients (85%) in the FCH1 group (mean [SD], 9.12 [1.2] mg/dL [2.28 (0.3) mmol/L]) vs 14 of 25 patients (56%) in the MIBI1 group (mean [SD], 9.32 [0.36] mg/dL [2.33 (0.09) mmol/L]) (difference, 29%; 95% CI, 4%-51%). Figure 3 illustrates the case of a woman with positive FCH1-guided MIP leading to normocalcemia during follow-up.

### Secondary Outcomes

#### Second-Line Imaging

In the FCH1 group, 5 patients underwent MIBI2. Two MIBI2 scans were positive, 1 inconclusive, and 2 negative. One patient with positive MIBI2 underwent MIP followed by normocalcemia at 1 month. The other patient with positive MIBI2 underwent video-assisted thoracoscopy for ectopy followed by immediate normalization of calcemia after surgery. One patient had both FCH1 and MIBI2 results that were inconclusive. She underwent bilateral cervical exploration with resection of a left superior PTA leading to normocalcemia at 1 month. One patient with negative MIBI2 underwent BCE with resection of a PTA, leading to normocalcemia at 1 month. The second underwent MIP guided by an inconclusive FCH1 examination, resulting in successful PTA resection and normocalcemia at 1 month.

In the MIBI1 group, 8 of 10 patients who underwent FCH2 were scored positive. Among these 8 patients, 7 underwent MIP resulting in normocalcemia at 1 month in 6 patients. Follow-up was missing for 1 patient. One patient underwent BCE for bilateral PTA leading to normocalcemia. Two patients had negative FCH2 examination results: 1 underwent BCE with resection of 1 PTA leading to normocalcemia at 1 month, whereas the other did not undergo surgery (nephrologist refusal).

#### Histology of Lesions After Surgery

Overall, 66 specimens were explored during surgery, of which 7 were inspected visually but not resected because results did not correspond to parathyroid tissue. Histologic analysis of the other 59 resected specimens revealed 56 PTA, 1 parathyroid hyperplasia, 1 normal parathyroid, and 1 lymph node. Mean (SD) histologic parathyroid lesion size was 11.2 (8.6) mm, without any difference between the groups.

#### Lesion-Based Diagnostic Performances of First-Line Imaging

Sensitivity of FCH1 and MIBI1 was 82% (95% CI, 62%-93%) and 63% (95% CI, 42%-80%), respectively. Positive predictive value was 92% (95% CI, 72%-99%) and 100% (95% CI, 77%-100%) for FCH1 and MIBI1, respectively.

#### Follow-Up

**Figure 4.**
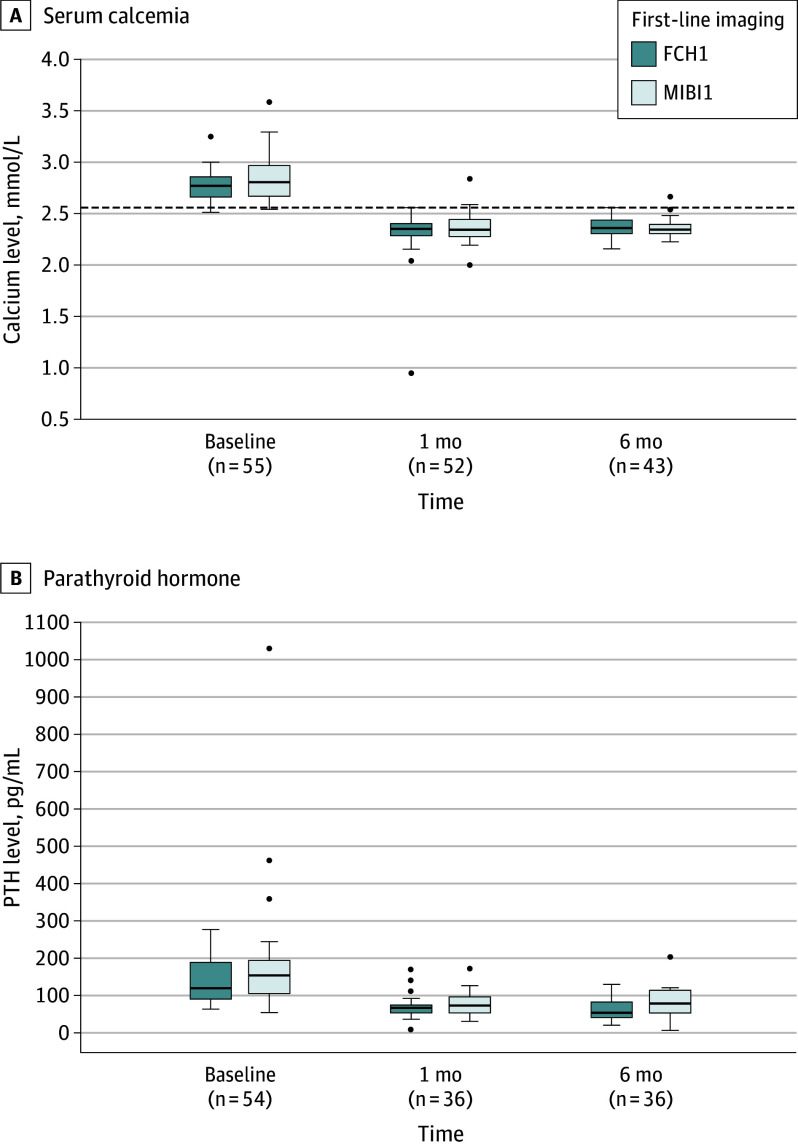
Laboratory Results at Baseline, 1 Month, and 6 Months FCH1 indicates first-line F18-choline (FCH) positron emission tomographic (PET)/computed tomographic (CT) scan PET/CT. Boxplots represent median, first and third quartiles, minimum (0 quartile) and maximum (fourth quartile) excluding outliers; the dots indicate outliers.

Follow-up at 1 and 6 months with biochemical measures was available in 52 (mean [SD] calcium levels, 9.28 [0.96] mg/dL [2.32 (0.24) mmol/L]) and 43 patients (mean [SD] calcium levels: 9.28 [0.96] mg/dL [2.36 (0.10) mmol/L]), respectively (Figure 4). All patients with normocalcemia at 1 month after surgery still had it at 6 months. One patient with negative MIBI1 and positive FCH2 did not attend the 1-month follow-up but had normocalcemia 6 months after MIP. One patient had persistent hypercalcemia 1 and 6 months after surgery despite a positive MIBI1 examination showing a left inferior PTA, which was successfully resected by MIP. The patient may thus be suspected of having multifocality missed by MIBI1.

#### Reporter Agreement

Patient-based reporter agreement (positive, negative, or inconclusive imaging results and localization of lesions) was almost perfect with FCH1 (κ = 0.81; 95% CI, 0.65-0.98) and MIBI1 (κ = 0.86; 95% CI, 0.71-1).

### Safety and Adverse Events

No adverse events associated with FCH administration were reported. Two transient adverse events related to surgery were observed: dysphonia and paresthesia of the arms and legs. Two transient adverse events related to general anesthesia were observed: acute urinary retention and hypotension with bradycardia.

## Discussion

The findings of this clinical trial show that FCH PET/CT can be used as a first-line imaging technique for preoperative PTA localization in the surgical management of PHPT, thereby suitably replacing MIBI SPECT/CT. We observed a superior rate of successful MIP leading to normocalcemia 1 month after surgery for FCH1 compared to MIBI1, a finding that can be explained by the higher sensitivity of FCH1 vs MIBI1. We also show that FCH PET/CT is safe for preoperative workup in both female and male patients with PHPT.

These findings are consistent with previous studies showing the diagnostic superiority of FCH PET/CT compared with MIBI SPECT/CT.^3,8,9^ However, our study focused on the clinical effect of first-line FCH PET/CT in contrast to most other studies focusing on FCH PET/CT in a second-line setting. The crossover design we used confirms the high yield of second-line FCH PET/CT, and shows that the yield of second-line MIBI SPECT/CT is lower.

### Strengths and Limitations

The study has several strengths. First, the multicenter randomized phase 3 design directly coupled diagnostic accuracy with effective therapy, allowing for the assessment of the clinical utility of upfront FCH PET/CT, information that cannot be obtained from a diagnostic cohort study design.^16^ Second, unlike studies focusing on diagnostic end points such as test accuracy, our focus was that of the patients, ie, being cured of PHPT by the least invasive procedures with a minimal risk of complications and satisfactory cosmetic results. Limiting the number of medical consultations is also a goal, especially when PHPT is asymptomatic. Our findings demonstrate the straightforwardness of up-front FCH PET/CT, leading most patients to successful MIP without the need for second-line imaging (Figure 4). In addition, the high precision of FCH PET/CT imaging allowed for almost perfect reporter agreement and an excellent localization of PTA during surgery.

Another strength is the multidisciplinary third look in the event of interrater discordance. In addition, we pursued the clinical pathway further than others by using the clinical parameter of calcemia 1 month after surgery as a surrogate for cure, whereas many other studies use histopathologic findings as a surrogate end point.

In contrast to the retrospective single-arm study of first-line FCH PET/CT by Broos et al,^17^ in our study we only included PHPT patients with a surgical indication who were fit enough and willing to undergo surgery. Except for 1, all 5 patients with double-negative or inconclusive imaging results underwent surgery (4 BCE and 1 MIP). By doing so, we aimed to reduce the selection bias frequently observed in other studies, ie, omitting patients with negative imaging results and patients without surgery from the calculation of diagnostic performance. This may explain why the sensitivity of FCH1 found in our study, 82%, was lower than the sensitivity higher than 90% reported in pooled data extracted from individual studies,^8,9,18,19^ although the upper bound of the confidence interval around the sensitivity value was 93%, and thus our results are compatible with the values from the published literature. Previously reported diagnostic performances of FCH PET/CT may thus be overestimated because they were calculated exclusively on patients with positive imaging results undergoing surgery. Furthermore, our study findings show that the outcome of BCE in experienced hands was excellent.

Some limitations of the study are the small sample size and the absence of follow-up beyond 6 months after surgery. Normalization of PTH levels was not a primary aim because they sometimes remain elevated even when long-term normocalcemia is obtained after successful parathyroidectomy.^20^ It might have been preferable to use corrected calcium levels, although albumin levels at baseline were normal in our study population. From a methodological point of view, the fact that the primary end point was assessed after second-line imaging could be criticized: it would have indeed been preferable and more robust to consider a study design without second line imaging, meaning that all patients with negative/inconclusive first-line imaging results would have undergone a BCE. Such considerations did not seem ethical for treatment of patients, given the results of our previous APACH1 study.^5^ Another potential limitation is that the raters were not blinded for the ultrasonography data performed before randomization, although this is common clinical practice that was followed in both study arms. Comparison of FCH PET/CT to other imaging techniques used for PTA detection such as ultrasonography, 4-dimensional contrast enhanced CT or magnetic resonance imaging was beyond the scope of our study. However, the network multimodality meta-analysis by Lee et al^9^ suggests the superiority of FCH PET/CT compared to all other imaging tests.

## Conclusions

This randomized clinical trial in patients with PHPT needing surgery demonstrates that first-line FCH PET/CT instead of MIBI SPECT/CT may be superior and safe for preoperative PTA localization, leading to successful MIP and normocalcemia 1 month after surgery. We expect these findings to contribute to the marketing authorization of this existing radiopharmaceutical for a new clinical indication.

## Notes

Supplement 1. Trial Protocol

Supplement 2. eMethods. Imaging Procedures

Supplement 3. Data Sharing Statement
