# Supplementary material for: F18-Choline PET/CT or MIBI SPECT/CT in the Surgical Management of Primary Hyperparathyroidism: A Diagnostic Randomized Clinical Trial
Source: JAMA Otolaryngol Head Neck Surg. 2024 Jun 20;150(8):658–65. doi: 10.1001/jamaoto.2024.1421 (PMC11190825; doi:10.1001/jamaoto.2024.1421)
Supplement: Supplement 1. — Trial Protocol [file jamaotolaryngolheadnecksurg-e241421-s001.pdf]

APACH2 TRIAL

**Phase III Trial Comparing 2 Diagnostic Strategies for Preoperative Localization of Parathyroid Adenoma in Primary Hyperparathyroidism: First-line SPECT/CT with Tc99m-sestaMIBI or PET/CT with F18-choline**

N°Eudract : 2019-000274-36

Version n°3.1 dated from 04/07/2021

This trial is granted by the ***Fondation de l'Avenir***

**REGLEMENTARY CLASSIFICATION OF TRIAL : Research involving human subjects of category 1**

**SPONSOR**	**Centre François Baclesse**<br>3 avenue du Général Harris<br>14076 CAEN cedex 5<br>Tél. : 02 31 45 50 50 – Fax : 02 31 45 51 58

**COORDONNATOR INVESTIGATOR**	**Dr Elske QUAK**<br>Centre François Baclesse<br>3 avenue du Général Harris<br>14076 CAEN cedex 5<br>E-mail: e.quak@baclesse.unicancer.fr<br>Tel.: 02 31 45 50 02 – Fax: 02 31 45 51 58

**COMPETENT AUTHORITY**	French Health Agency (ANSM)	Authorisation date: 25/06/2019<br>Amendment 1 authorised on 06/05/2020<br>Amendment 2 authorised on 20/04/2021
**ETHIC COMMITTEE**	Ethics committee SOUTHEAST II	Initial approval : 26/07/2019<br>Amendment 1 approved on 16/06/2020<br>Amendment 2 approved on 29/04/2021

CONFIDENTIALITY STATEMENT

The information contained in this document is the property of Centre François Baclesse and therefore is provided to you in confidence for review by you, your team, an applicable Ethics Committee/Institutional Review and regulatory authorities. It is understood that the information will not be disclosed to third parties without prior written approval from Centre François Baclesse, except to the extent necessary to obtain informed consent from those persons to whom the medication may be administered.

PEOPLE INVOLVED IN THE PREPARATION AND THE CONDUCT OF THE PROTOCOL

INVESTIGATOR CENTERS		
**CENTER NO.**	**INVESTIGATORS**	**LOCATION OF THE RESEARCH**
**1**	**Principal Investigator:**<br>Dr. Elisabeth QUAK, MD<br>**Co-investigator:**<br>Dr. Stéphane BARDET, MD<br>Dr. Renaud CIAPPUCCINI, MD<br>Dr. Charline LASNON, MD<br>Dr. Audrey LASNE-CARDON MD<br>Prof. Emmanuel BABIN, MD, PhD<br>Dr. Vianney BASTIT, MD	**Comprehensive Cancer Centre François Baclesse (CFB), CAEN**
**2**	**Principal Investigator:**<br>Dr. Marie CAVAREC, MD<br>**Co-investigator:**<br>Prof. Pierre-Yves SALAUN, MD, PhD<br>Dr Nathalie KEROMNES, MD<br>Dr. Nathalie ROUDAUT, MD<br>Dr. Gael POTARD, MD	**BREST University Hospital**
**3**	**Principal Investigator:**<br>Dr. Anne DEVILLERS, MD<br>**Co-investigator:**<br>Dr. Antoine Girard MD<br>Dr. Marie Luce Barge MD<br>Dr. Cécile CuvilliersMD<br>Dr. Guery Clemence MD<br>D Lenoir Laurence MD<br>Dr. Palard Xavier MD	**Comprehensive Cancer Centre Eugène Marquis, RENNES**
**4**	**Principal Investigator:**<br>Prof. Franck JEGOUX, MD, PhD<br>**Co-investigator:**<br>Dr. Olivier CROUY-CHANEL, MD<br>Dr. Patricia VADUVA, MD	**RENNES University Hospital**

ASSOCIATED TEAMS	
**CLINICAL RESEARCH DEPARTMENT**	Centre François Baclesse – CAEN<br>Tel: +33 (0)2 31 45 50 02<br>Fax: +33 (0)2 31 45 51 58
**Promotion Manager:**<br>Bénédicte CLARISSE	b.clarisse@baclesse.unicancer.fr
**Project Manager**<br>Jean-Michel GRELLARD	jm.grellard@baclesse.unicancer.fr
**Methodologist:**<br>Justine LEQUESNE	j.lequesne@baclesse.unicancer.fr
**Pharmacovigilance**<br>Marie CASTERA-TELLIER	m.castera@baclesse.unicancer.fr
**Medico-economist**<br>Celia BERCHI	celia.berchi@unicaen.fr

TABLE DES MATIERES

1 SYNOPSIS 5
2 STUDY OVERVIEW 8
3 STUDY FLOW CHART 9
4 SCIENTIFIC RATIONALE OF THE STUDY 10
4.1 BACKGROUND 10
4.2 ORIGINALITY AND INNOVATION 10
4.3 EXPECTED BENEFIT TO THE PATIENT 11
5 STUDY OBJECTIVES 11
5.1 PRIMARY OBJECTIVE 11
5.2 SECONDARY OBJECTIVES 11
6 ENDPOINTS 11
6.1 PRIMARY ENDPOINT 11
6.2 SECONDARY ENDPOINTS 12
7 STUDY DESIGN 12
7.1 METHODOLOGY 12
7.2 STUDY DURATION 12
7.3 SUBJECTS SELECTION 12
7.3.1 Inclusion criteria 12
7.3.2 Non inclusion criteria 12
7.4 STUDY PLAN 13
7.4.1 Consent sign 13
7.4.2 Randomization procedure 13
7.4.3 Study conduct 13
7.4.4 Methods and Techniques Used or Evaluated in the Study 14
7.4.5 Precautionary Principles in Women 15
7.4.6 Dosimetry 15
8 PHARMACEUTICAL FORM OF STUDY MEDICATIONS 16
8.1 MIBI 16
8.1.1 Introduction to the RPM 16
8.1.2 Method of administration 16
8.1.3 Labelling 16
8.1.4 RPM Circuit for the Centre François Baclesse 16
8.1.5 RPM Circuit for Brest University Hospital 17
8.1.6 RPM Circuit for the Centre Eugène Marquis 17
8.2 FCH 17
8.2.1 Presentation of the RPM 17
8.2.2 Method of administration 17
8.2.3 Labelling 17
8.2.4 RPM Circuit for the Centre François Baclesse 18
8.2.5 RPM Circuit for Brest University Hospital 18
8.2.6 RPM Circuit for the Centre Eugène Marquis 18
9 MEDICO-ECONOMIC STUDY 19
10 SAFETY 19
10.1 GENERAL RULES – INSTRUCTIONS 19
10.2 DEFINITIONS 19
10.2.1 Adverse Event (AE) 19
10.2.2 Adverse Reaction 19
10.2.3 SERIOUS Adverse event 19
10.2.4 Suspected Unexpected Adverse Reaction 20
10.3 INVESTIGATOR'S RESPONSIBILITIES 20

10.3.1 Detection and registration of adverse events *20*
10.3.2 SAE reporting process *21*
10.3.3 Special cases *22*

11 STATISTICAL CONSIDERATIONS 22

11.1 Sample size 22
11.2 Statistical analysis 23

12 QUALITY CONTROL 23

13 ETHICS AND REGULATORY CONSIDERATIONS 23

13.1 Clinical trial authorisation 24
13.2 Information of patients involved in the research 24
13.3 Investigator responsibilities 24
13.4 Data confidentiality 25

14 DATA AND DOCUMENTS KEEPING 25

14.1 Data entry and handling 25
14.2 Archiving 26
14.3 Publication policy 26

15 FUNDING AND INSURANCE 26

15.1 Funding 26
15.2 Insurance 26

16 REFERENCES 27

1 SYNOPSIS

**TITLE**	**Phase III Trial Comparing 2 Diagnostic Strategies for Preoperative Localization of Parathyroid Adenoma in Primary Hyperparathyroidism: First-line SPECT/CT with Tc99m-sestaMIBI or PET/CT with F18-choline**
**ACRONYM**	**APACH-2**
**Coordinator**	**Dr Elske QUAK**
**Indication**	**Patient with primary hyperparathyroidism and for whom excision surgery is planned.**
**Design**	**Phase III, randomized, open-label, multicenter study**
**Objectives**	**Main objective**<br>To compare, between each diagnostic strategy, the proportion of patients for whom the **first-line** imaging technique (MIBI SPECT/CT or FCH PET/CT) was used to guide the surgical procedure appropriately (true positive minimally invasive surgery decision and patient cure)<br>**Secondary objectives**<br>- To conduct a medico-economic evaluation comparing the costs and effectiveness of the two diagnostic strategies<br>- To estimate the diagnostic performance of each strategy<br>- To assess the number of failures at 6 months of parathyroid surgery (regardless of the type of surgery performed) for each strategy<br>- To assess post-surgical complications<br>- To assess intra- and inter-observer variability for the interpretation of FCH PET/CT and MIBI SPECT/CT<br>- To explore the relationship between the positivity of imaging tests, including FCH PET/CT and serum PTH concentration at baseline<br>- To evaluate the detection performance of FCH PET/CT at an early imaging time point at 10 min post-injection compared to that of 60 min post-injection.<br>- To assess patient satisfaction in the perioperative period
**Judgement criteria**	**Main criterion**<br>Proportion of patients for whom **first-line** imaging technique (MIBI SPECT/CT or FCH PET/CT) guided the surgical procedure appropriately as defined by:<br>- A decision to perform a truly positive (wise) minimally invasive surgery<br>○ **AND**<br>- Achieving a cure for the patient, defined as normalization of serum calcium and PTH at one month and 6 months post-surgery<br>**Secondary criteria**<br>- Average cost of caring for a patient for each of the two strategies<br>- Sensitivity, specificity, error rates, positive and negative likelihood ratios of PET-CT AT CHF and SPECT/CT at MIBI<br>- Rate of minimally invasive surgeries and bilateral cervical explorations for each diagnostic strategy<br>- Number of failures of parathyroid surgery (regardless of the type of surgery performed), defined by the persistence of primary hyperparathyroidism 6 months after surgery<br>- Complications occurring within one month of surgery: infections, hematomas, laryngeal recurrent nerve injury<br>- Cohen's kappa coefficients between the 2 independent readers, during their

	first reading, then during their second reading (3 months later); as well as between the first and second reading of each of the 2 readers<br>- Serum PTH concentration at baseline<br>- To compare detection sensitivities of FCH PET-CT at 10 min and 60 min post-injection, as well as semi-quantitative analysis.<br>- Scores for the 6 dimensions (comfort, attention, information, pain management, waiting times, intimacy) of the EVAN-G self-questionnaire administered perioperatively prior to hospital discharge
**Inclusion criteria**	- Patient over 18 years of age<br>- Patient with primary hyperparathyroidism and for whom excision surgery is planned<br>- For women of childbearing potential, negative pregnancy test at baseline<br>- Laboratory assessment confirming the diagnosis of primary hyperparathyroidism (elevated serum PTH and calcium concentrations)<br>- Affiliation to a social security scheme<br>- Patient who has signed written consent
**Non inclusion criteria**	- Patient deprived of liberty, under guardianship or curatorship<br>- Hypersensitivity to TECNESCAN SESTAMIBI<br>- Any associated medical or psychological condition that could compromise the patient's ability to participate in the study<br>- Pregnant or breastfeeding woman<br>- History of parathyroid surgery<br>- Patient with multiple endocrine neoplasia 1 (MEN1)<br>- Known hypersensitivity to Fluorocholine or any of the excipients (sodium chloride, water for injections)
**Experimental Plan**	Eligible patients will be randomized to one of two strategies:<br>• **<u>Standard arm</u>**: MIBI SPECT/CT and then, if negative, FCH PET/CT scan.<br>• **<u>Experimental arm</u>**: FCH PET/CT scan, and then, in case of negativity, a MIBI SPECT/CT.<br>All examinations will be performed within 12 weeks prior to parathyroid surgery.<br><u>● Imaging</u><br>✓ <u>MIBI SPECT-CT</u><br>The patient will receive 740 MBq of MIBI intravenously. Ten minutes post-injection, an early planar image of the thyroid compartment will be performed. Ninety minutes post-injection, a SPECT acquisition as well as a low-dose CT scan will be acquired of the neck and mediastinum. The duration of early acquisition will be 10 minutes and 25 minutes for SPECT/CT.<br><br>✓ <u>FCH PET-CT</u><br>Installed in a radioprotected room, the patient will receive 1.5 MBq/kg of FCH as an intravenous infusion. Ten and sixty minutes post-injection, PET scan as well as a low-dose CT scan will be acquired of the neck and mediastinum in the supine position (total acquisition time of 10 minutes).<br><br>The interpretation of PET/CT and SPECT/CT examinations will consist of indicating the presence of focal points suggestive of PTA by specifying its location, its CT scan dimensions, its volume, and for the PET, the metabolic profile. For the same patient, each of the 2 examinations will be interpreted by 2 blinded different operators. In case of a match, the result will be sent to the surgeon. In the event of

	a discrepancy, the examinations will be reviewed in a Multidisciplinary Consultation Meeting (RCP), and the result of the review of the examination will be communicated to the surgeon.<br><br>Surgical decision for each diagnostic strategy<br>In case of positive results of the first and/or second exam, the surgery will consist of a minimally invasive procedure under extemporaneous, on an outpatient basis.<br>In case of negative imaging results (MIBI and FCH), an exploration of the 4 parathyroid sites will be performed.<br>In the case of multiple or ectopic PTAs, the surgical procedure will be adapted.<br><br>Histopathological examination of the surgical specimens will confirm the presence or absence of PTA.<br>The results of the imaging assessment will be compared with the surgical and pathological results.<br>Patients will be seen 1 and 6 months post-surgery with a blood test to assess normalization of serum calcium and PTH.
**Sample size**	Considering that the proportion of patients referred to the right surgical strategy according to first-line imaging is 60% with MIBI SPECT/CT [Cheung, 2012] and 90% with FCH PET-CT [Quak, 2017], 50 evaluable patients (25 per arm) are needed to detect such a difference in proportions with a risk of 5% and a power of 80% (unilateral test). It is planned to include 58 patients to anticipate 15% of possible non-evaluable patients.
**Participating centres**	4
**Duration of the study**	36 months (30 months inclusion and maximum 6 months follow-up)

2 STUDY OVERVIEW

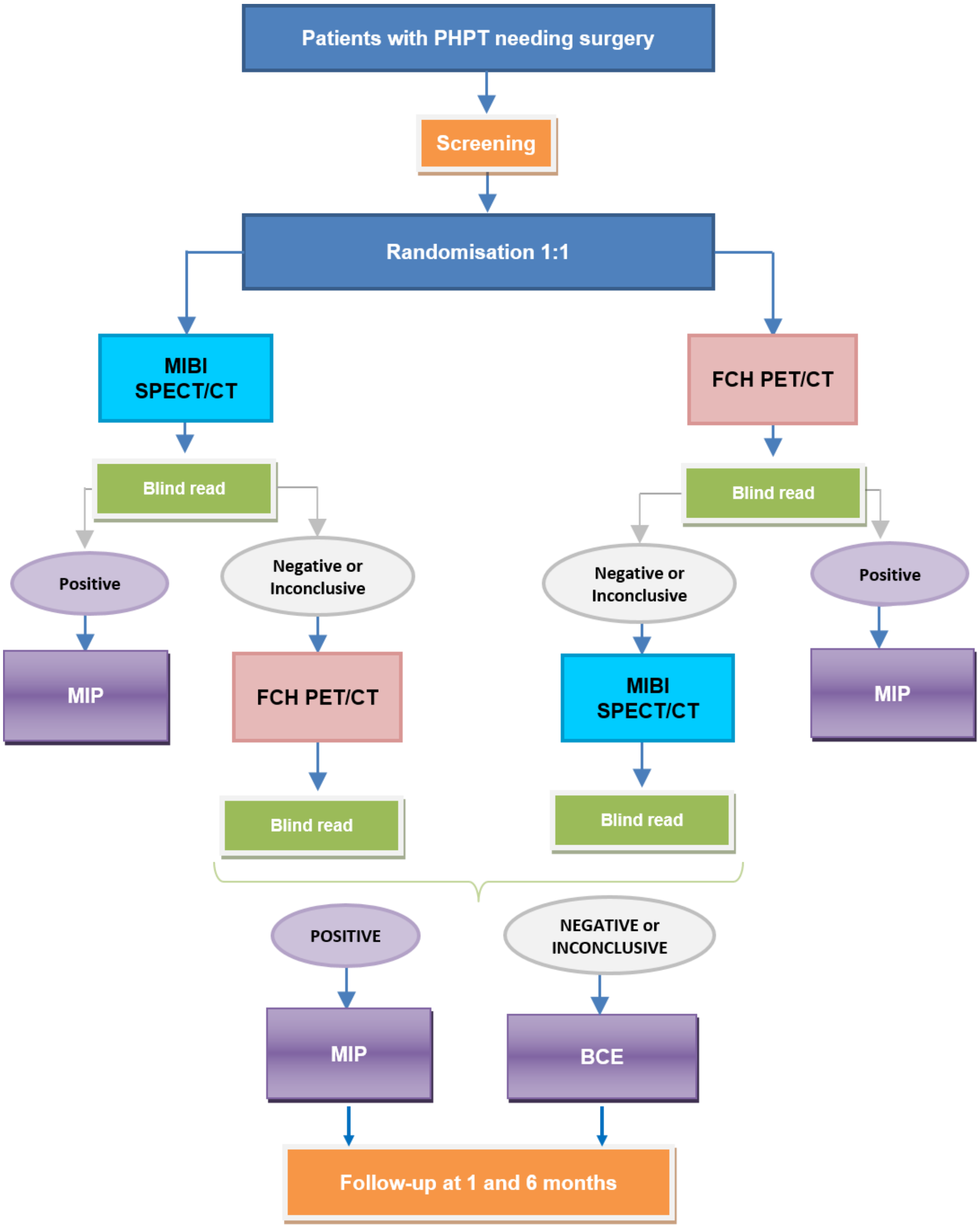

PHPT = primary hyperparathyroidism; MIP = minimally invasive parathyroidectomy; BCE = bilateral cervical exploration

3 STUDY FLOW CHART

	Before inclusion	Before Surgery	Surgery	At 1 and 6 months post-surgery
**Informed Consent**	□			
**History of the disease**	□			
**Physical examination (weight, height)**	□			
**Pregnancy test** (urine or blood) (within the previous 8 days)	□			
**Cervical ultrasound**	□			
**Blood tests***	□			□
**F18-choline PET-CT and/or MIBI SPECT-CT** (within 12 weeks prior to surgery; at least 48 hours between the 2 exams)		□		
**For patients undergoing a PET-choline examination, a telephone call the day after the examination to assess tolerance**		□		
**Adverse events**		□	□	□
**Patient satisfaction** (EVAN-G questionnaire)			□**	

* <u>Biochemistry</u>: *Calcium, phosphoremia, albumin, creatinine, PTH, 25 OH vitamin D*

**completed within 4 to 48 hours after surgery, before discharge from hospitalization

4 SCIENTIFIC RATIONALE OF THE STUDY

4.1 BACKGROUND

Primary hyperparathyroidism is a common endocrine disease, related to autonomic overproduction of parathyroid hormone (PTH), usually by a parathyroid adenoma (PTA). The diagnosis is confirmed by a laboratory test. Excision of PTA remains the only curative treatment: despite a diagnosis often at an asymptomatic stage, normalization of hypercalcemia by excision of PTA improves bone mineral density and quality of life of patients. Bilateral cervical exploration of the parathyroids is now supplanted by minimally invasive surgery requiring precise localization of the PTA preoperatively. This minimally invasive technique, usually performed on an outpatient basis under local anesthesia, is associated with a better cure rate and fewer complications than bilateral cervical exploration, and at a lower cost (Udelsman-2011).

The current diagnosis to localize PTA is based on Tc99m-sestaMIBI (MIBI) scintigraphy with tomographic acquisition and CT scanning, negative in 33% of cases, leading to the proposal of bilateral cervical exploration (Ciappuccini-2012).

The recent literature and the results of our prospective APACH1 study (NCT02432599) strongly suggest the best diagnostic value of positron emission tomography coupled with F18-choline (FCH) CT scanning. Out of 25 patients with primary hyperparathyroidism and a negative MIBI scan, second-line FCH PET/CT was positive in 19 patients guiding parathyroidectomy and successfully reduced operative time in the majority of patients (Quak-2017).

Although FCH PET/CT is not yet used as a first-line treatment for primary hyperparathyroidism (cost of examination, availability of PET cameras, lack of marketing authorization for FCH in this indication), its contribution to guiding the surgical procedure seems promising. It therefore seems interesting to question the place of FCH PET-CT as a first-line imaging technique to localize PTA in biologically confirmed primary hyperparathyroidism, while taking into account the costs.

The delay to perform PET-CT images after FCH injection varies depending on the team (from 0 to 2 hours, some favouring 30 min images, others 60 min and others two acquisitions at 5 and 60 min) (Treglia-2018). Few studies have compared two acquisition times, one early (5-15 min) and the other later (60 min). A retrospective study conducted on 43 patients shows better contrast from 60 min post-injection (Rep-2015). Another study describes an increase in SUVmax at late time on a preliminary study, but data are limited (Thanseer-2017). However, other authors have found good performance in <15 min imaging (Michaud-2014; Prabhu 2018). The advantage of early imaging after injection would be to avoid a waiting time of one hour in an injection box for the patient and to allow better management of the organization of the PET program of a nuclear medicine department. It therefore seems interesting to prospectively evaluate two PET-CT imaging times at the FCH and to compare them in terms of detection sensitivity and semi-quantitative analysis.

4.2 ORIGINALITY AND INNOVATION

In accordance with the recommendations on the methodology of evaluating diagnostic techniques (Rodger, Trials, 2012), we propose the first prospective randomized study comparing 2 diagnostic strategies (MIBI SPECT/CT or FCH PET/CT) first-line for the preoperative localization of PTA.

We seek to evaluate the best first-line diagnostic strategy to localize PTAs and guide surgery, at the lowest cost to society, by comparing the 2 strategies in terms of efficacy and cost of each strategy.

We hypothesize that FCH PET/CT will reduce the proportion of unnecessary invasive surgery decisions and that the higher cost of PET scan compared to MIBI will be offset by a lower cost in terms of the type of surgery performed correctly and complications.

4.3 EXPECTED BENEFIT TO THE PATIENT

Insofar as the only treatment with curative aim remains surgical excision of the PTA, the challenge of being able to locate the PTA prior to the surgical procedure is major to guide the surgery. While minimally invasive surgery, associated with more cures and fewer complications, as well as lower cost, is preferred, it is only possible if the PTA is previously localized. However, to date, MIBI scintigraphy combined with SPECT/CT is not contributory for a third of patients, for whom bilateral cervical exploration is therefore proposed.

We hypothesize that first-line FCH PET/CT will reduce the proportion of unnecessary invasive surgery decisions and that the higher cost of PET scanning compared to MIBI will be offset by a lower cost in terms of the type of surgery performed appropriately and complications. If we test this hypothesis by prospectively randomizing two diagnostic strategies, as recommended to evaluate diagnostic techniques (Rodger, Trials, 2012), the use of FCH PET/CT as a first-line treatment to localize PTA could be considered to improve the therapeutic management of patients, at a lesser cost to the community.

5 STUDY OBJECTIVES

5.1 PRIMARY OBJECTIVE

The primary objective is to compare, between each diagnostic strategy, the proportion of patients for whom the first-line imaging technique (MIBI SPECT/CT or FCH PET/CT) guided the surgical procedure appropriately (true positive minimally invasive surgery decision and patient cure).

5.2 SECONDARY OBJECTIVES

The secondary objectives are to:

- Conduct a medico-economic evaluation comparing the costs and effectiveness of the two diagnostic strategies
- Estimate the diagnostic performance of each strategy
- Assess the number of failures at 6 months of parathyroid surgery (regardless of the type of surgery performed) for each strategy
- Assess post-surgical complications
- Assess intra- and inter-observer variability for the interpretation of FCH PET/CT and MIBI SPECT/CT
- Explore the relationship between the positivity of imaging tests, including FCH PET/CT, and serum PTH concentration at baseline
- Evaluate the detection performance of FCH PET-CT on an early imaging time point at 10 min post-injection compared to that of acquisition at 60 min.
- Assess patient satisfaction in the perioperative period

6 ENDPOINTS

6.1 PRIMARY ENDPOINT

The primary endpoint of the study is the proportion of patients for whom the **first-line** imaging technique (MIBI SPECT/CT or FCH PET/CT) was able to guide the surgical procedure appropriately defined by:

- A decision to perform a truly positive (wise) minimally invasive surgery

  AND

- Achieving a cure for the patient, defined as normalization of serum calcium at one month post-surgery

6.2 SECONDARY ENDPOINTS

The secondary endpoints are:

- Average cost of caring for a patient for each of the two strategies
- Sensitivity, specificity, error rates, positive and negative likelihood ratios of FCH PET/CT and MIBI SPECT/CT
- Rate of minimally invasive surgeries and bilateral cervical explorations for each diagnostic strategy
- Number of failures of parathyroid surgery (regardless of the type of surgery performed), defined by the persistence of primary hyperparathyroidism 6 months after surgery
- Complications occurring within one month of surgery: infections, hematomas, laryngeal recurrent nerve injury
- Cohen's kappa coefficients between the 2 independent readers, during their first reading, then during their second reading (3 months later); as well as between the first and second reading of each of the 2 readers
- Serum PTH concentration at baseline
- To compare detection sensitivities of FCH PET-CT at 10 min and 60 min post-injection, as well as semi-quantitative analysis.
- Scores for the 6 dimensions (comfort, attention, information, pain management, waiting times, intimacy) of the EVAN-G questionnaire administered perioperatively (4 to 48 hours after the intervention before discharge from hospitalization)

7 STUDY DESIGN

7.1 METHODOLOGY

We propose a phase III, randomized, open-label, multicenter study comparing, between each diagnostic strategy, the proportion of patients for whom the first-line imaging technique (MIBI SPECT/CT or FCH PET/CT) was used to guide the surgical procedure wisely (true positive minimally invasive surgery decision and patient cure).

7.2 STUDY DURATION

The expected duration of the recruitment is 30 months.

The duration of the follow-up period is 6 months post-surgery.

7.3 SUBJECTS SELECTION

7.3.1 Inclusion criteria

- Patient over 18 years of age
- Patient with primary hyperparathyroidism and for whom excision surgery is planned
- Laboratory assessment confirming the diagnosis of primary hyperparathyroidism (elevated serum PTH and calcium concentrations)
- For women of childbearing potential, negative pregnancy test at baseline
- Affiliation to a social security scheme
- Patient who has signed written consent

7.3.2 Non inclusion criteria

- Patient deprived of liberty, under guardianship or curatorship
- Hypersensitivity to TECNESCAN SESTAMIBI

- Any associated medical or psychological condition that could compromise the patient's ability to participate in the study
- Pregnant or breastfeeding woman
- History of parathyroid surgery
- Patient with MEN1
- Known hypersensitivity to Fluorocholine or any of the excipients (sodium chloride, water for injections)

7.4 STUDY PLAN

7.4.1 Consent sign

The study will be proposed by the investigators to patients who meet the eligibility criteria. They will be given an information note and an informed consent form. Patients will have a reflection period before choosing to participate.

After obtaining the patient's consent by signing the study consent, the selection criteria will be checked prior to inclusion in the trial.

Study-specific examinations requested prior to inclusion (inclusion assessment) will be performed after signing consent and prior to randomization.

7.4.2 Randomization procedure

After obtaining the patient's consent by signing the study consent, verifying the selection criteria and carrying out the inclusion assessment, randomization will be carried out.

Randomization will be performed on software dedicated to the study via a web portal.

Eligible patients will be randomized to one of two strategies:

•Standard arm: MIBI SPECT/CT and then, if negative, FCH PET/CT scan.

•Experimental arm: FCH PET/CT scan, and then, in case of negativity, a MIBI SPECT/CT.

An identification number will be assigned to the patient and will be used throughout the study.

7.4.3 Study conduct

❖ **Inclusion Assessments**

Patients who are eligible for the trial and who have signed their consent to participate will be required to undergo a clinical examination and laboratory assessment prior to randomization as follows:

Relevant history (risk factors, associated pathologies, medical history and concomitant treatments) as well as the history of the disease and current treatments should be reported.

The physical examination should include weight and height.

A pre-therapeutic laboratory assessment should be carried out and will include: calcemia, phosphoremia, albumin, creatinine, PTH, 25 OH vitamin D.

Neck ultrasound.

These examinations correspond to those usually performed as part of the patient's routine management, in addition to MIBI SPECT/CT.

As part of the protocol, women of childbearing potential will be asked to take a urine or biological pregnancy test within 8 days prior to imaging.

❖ **Protocol Imaging Assessment**

FCH PET-CT and/or MIBI SPECT/CT will be performed within 12 weeks prior to parathyroid surgery. The 2 exams will be at least 48 hours apart.

For women who have undergone a PET exam with Fluorochol®, they will be contacted by telephone the day after the examination: this protocol precaution is put in place to ensure the absence of adverse effects, in accordance with the rapid biokinetics and elimination of Fluorochol® (approximately 12 hours).

Before performing these two examinations, the patient will be asked to fast for at least 4 hours before the injection without restriction of fluid intake.

The doctor will also check that the patient is not taking any contraindicated treatments with the imaging tests.

❖ **Parathyroid Surgery**

It will be performed no later than 12 weeks after the last scintigraphic examination (MIBI SPECT/CT or FCH PET/CT).

Depending on the results of the imaging tests, the surgical procedure will consist of minimally invasive surgery or bilateral cervical exploration. The sample will be sent for extemporaneous examination during surgery.

A nasofibroscopic check-up will be performed postoperatively before discharge from hospitalization to assess the mobility of the vocal cords.

Patient satisfaction in the perioperative period will be assessed using the EVAN-G questionnaire within 48 hours of the procedure.

❖ **Follow-up at 1 and 6 months after surgery**

A laboratory assessment including calcemia and PTH will be performed at 1 and 6 months after surgery.

7.4.4 Methods and Techniques Used or Evaluated in the Study

❖ **Imaging**

→ MIBI SPECT/CT

The patient will receive 740 MBq of MIBI intravenously. Ten minutes post-injection, an early planar image of the thyroid compartment will be performed. Ninety minutes post-injection, a SPECT scan centered on the neck and mediastinum as well as a low-dose CT scan will be acquired. The lenses will not be included in the acquisition field: the scanner will start under the eye sockets. The duration of early acquisition will be 10 minutes and 25 minutes for SPECT/CT.

→ FCH PET/CT

The patient will receive 1.5 MBq/kg FCH as an intravenous infusion in an injection box. Ten and sixty minutes post-injection, PET images centered on the neck and upper mediastinum as well as a low-dose CT scan will be acquired (total acquisition time of 10 minutes, corresponding to 2 steps of 5 minutes each). The lenses will not be included in the acquisition field: the scanner will start under the eye sockets.

The interpretation of PET/CT and SPECT/CT examinations will consist of indicating the presence of focal points suggestive of PTA by specifying its location, its CT scan dimensions, its volume, and for the PET, the metabolic profile.

For the same patient, each of the 2 examinations will be interpreted by 2 different operators in a blinded fashion. In case of a match, the result will be sent to the surgeon. In the event of a discrepancy, the examinations will be reviewed in a Multidisciplinary Consultation Meeting (RCP), and the result of the review of the examination will be communicated to the surgeon.

❖ **Surgical decision for each diagnostic strategy**

In case of positive results of the first and/or second exam, the surgery will consist of a minimally invasive procedure, on an outpatient basis.

In case of negative imaging results (MIBI and FCH), an exploration of the 4 parathyroid sites will be performed.

In the case of multiple or ectopic PTAs, the surgical procedure will be adapted.

❖ **Histopathological examination of surgical specimens**

Histopathological examination of the surgical specimens will confirm the presence or absence of PTA.

The results of the imaging assessment will be compared with the surgical and pathological results.

Patients will be seen again between 1 and 6 months post-surgery with a blood test to assess normalization of serum calcium.

❖ **Patient satisfaction in the perioperative period**

It will be assessed within 4 to 48 hours after surgery, before discharge, using the EVAN-G questionnaire (Auquier, Anesthesiology, 2005). This is a standardized and validated questionnaire, the purpose of which is to understand the experience of the perioperative period in a multidimensional way. It consists of 26 items structured in 6 dimensions called "Attention, Information, Intimacy, Pain, Comfort and Waiting Times". A global index is calculated as the sum of the dimensions. The scores of the dimensions as well as the overall index range from 0 (worst possible experience) to 100 (best possible experience).

7.4.5 Precautionary Principles in Women

Since Fluorochol® and CISCholine® are not indicated for use in women, eligible women will be required to take a pregnancy test prior to the examination, which must be negative to meet the eligibility criteria.

In addition, any patient who has undergone a PET-choline examination with Fluorochol or CISCholine will be contacted the day after the examination by telephone: this protocol precaution is put in place to ensure the absence of adverse effects, in accordance with the biokinetics and rapid elimination of Fluorochol® or CISCholine® (approximately 12 hours).

7.4.6 Dosimetry

The radiation reduction strategy is specified below during each of the two imaging examinations under study. Only patients for whom the first-line examination is negative or non-contributory will perform the 2 imaging examinations.

For FCH PET, the injected activity will be reduced from 3.5 MBq/Kg (standard dose for a FCH PET examination in oncology) to 1.5 MBq/kg, or approximately 105 MBq for a 70 kg patient. In return, the acquisition time will be increased to 5 minutes per step in list mode. According to the FCH dosimetry literature, the effective dose received is 0.018 mSv per MBq injected (Giussani, JNM 2012; ICRP, 2013). The irradiation associated with the injection of FCH in our study is therefore estimated at 1.89 mSv.

For the MIBI examination, the injected dose for the SPECT examination is 750 MBq of Sestamibi labelled with 99mTc. The effective dose received is 0.009mSv/MBq (see MA). The irradiation related to the injection for the SPECT is therefore 6.75mSv.

Each examination will be focused on the neck and upper mediastinum and the lenses will be excluded from the volume, in order to limit X-ray exposure. The dose-length product of a consecutive series of 31 patients of variable morphotype (body mass index range 15.4 to 38.1,

median 25.1) who underwent ENT-centered PET-CT in our PET unit was 150.6 ± 3.54 mGy.cm, the expected CT dosimetry will be 0.89 ± 0.02 mSv.

For the SPECT/CT, the dose received during the CT is about 50% more due to a 2-slice system instead of 64 on the CT of the PET. The dose received during the CT scan of the gamma camera is then of the order of 1.33mSv. The total effective dose of the two CT scans is in the order of 2.23mSv.

The total cumulative effective dose is therefore estimated to be 10.87 mSv.

Radiation protection measures for patients : As a precautionary measure, it is recommended that patients stay away from pregnant women and young children for an extended period of time for 12 hours after injection.

8 PHARMACEUTICAL FORM OF STUDY MEDICATIONS

8.1 MIBI

8.1.1 Introduction to the RPM

Technetium-99 m-labelled Sestamibi (2-methoxy-isobutyl-isonitrile) is in the form of a radiopharmaceutical preparation kit, to be labelled with sodium pertechnetate (Tc99m) solution for injection. 99mTc period: 6.02 hours. Gamma-ray single-photon peak of 140 keV.

8.1.2 Method of administration

Technetium-99-metastable labeled with sestamibi will be administered intravenously.

8.1.3 Labelling

The dispensing label will include the following information:

- the name of the radiopharmaceutical preparation: 99mTc-sestamibi
- Study reference: APACH2
- the batch number;
- the activity;
- the volume;
- the exact date and time of measurement at the end of the preparation;
- the exact expiry date and time of use;
- the symbol indicating the presence of radioactive substances ("standard trisector").

8.1.4 RPM Circuit for the Centre François Baclesse

- ✓ Reception: The Sestamibi® will be delivered, unmarked, to the airlock of the radiopharmacy of the François Baclesse Centre.
- ✓ Storage: The Sestamibi® should be stored in the refrigerator at 4°C
- ✓ Preparation: Preparation will be carried out in the low-energy leaded chamber, on the premises of the radiopharmacy (class D controlled atmosphere area, in accordance with Good Preparation Practices). The marking of Sestamibi®, using a 99mTc solution, will be carried out extemporaneously by a MER on the morning of the examination. A quality control will be carried out by the radiopharmacist to assess the quality of the marking. The product will be released if the Radiochemical Purity (PRC) is greater than 95%
- ✓ Method of dispensing: Once the preparation has been released by the radiopharmacist, the MER will prepare the patient's syringe taking into account the prescribed activity and the time of examination. The traceability of the dose (batch number, prescription number, patient identification, dose dispensed, dose administered, expiry date, etc.) will be carried out on the VENUS® nuclear medicine software (Nicesoft).
- ✓ Accounting allowing an assessment of compliance (accounting form): Given the radioactive nature of 99mTc-Sestamibi® (physical period 6.02h) and the single injection into the

department, compliance will be evaluated thanks to the traceability carried out on the nuclear medicine software VENUS® (Nicesoft).

8.1.5 RPM Circuit for Brest University Hospital

- ✓ Reception: The Sestamibi® kit will be delivered to the reception room of the nuclear medicine department of the Morvan Hospital of the Brest University Hospital
- ✓ Storage: The Sestamibi® kit will be stored at room temperature
- ✓ Preparation: The preparation will be carried out in the low-energy leaded chamber, on the premises of the radiopharmacy (class D controlled atmosphere area, in accordance with Good Preparation Practices). The marking of Sestamibi®, using a 99mTc solution, will be carried out extemporaneously by a MER on the morning of the examination. A quality control will be carried out by the laboratory technician or related technician, in order to evaluate the quality of the marking. The product will be released by the radiopharmacist if the Radiochemical Purity (PRC) is greater than 94%
- ✓ Method of dispensing and traceability: Once the preparation has been released by the radiopharmacist, the MER will prepare the patient's syringe taking into account the prescribed activity and the time of examination. The traceability of the dose (batch number, prescription number, patient identification, dose dispensed, dose administered, expiry date, etc.) will be carried out on the GERA® (Theleme) nuclear medicine business software in the first phase, followed by XPLORE® (EDL) in a second phase.

8.1.6 RPM Circuit for the Centre Eugène Marquis

- ✓ Reception: The Sestamibi® kit will be delivered to the reception room of the nuclear medicine department of the Eugène Marquis Centre in Rennes
- ✓ Storage: The Sestamibi® kit will be stored in a refrigerated 4°C-8°C chamber with continuously controlled temperature
- ✓ Preparation: Preparation will be carried out in the low-energy leaded enclosure, in the radiopharmacy premises (class D controlled atmosphere zone, in accordance with Good Preparation Practices). The marking of the Sestamibi®, using a 99mTc solution, will be carried out extemporaneously by a PPH or MER, the morning of the examination. A quality control will be carried out by a MER or a PPH, in order to assess the quality of the marking. The product will be released by the radiopharmacist if the Radiochemical Purity (PRC) is greater than 95%
- ✓ Dispensing method and traceability: Once the preparation has been released by the radiopharmacist, the MER or PPH will prepare the patient's syringe taking into account the prescribed activity and the examination time. Dose traceability (batch number, prescription number, patient identification, dose dispensed, dose administered, expiry date, etc.) will be carried out on the professional nuclear medicine software GERA® (Theleme).

8.2 FCH

8.2.1 Presentation of the RPM

The radionuclide fluorine-18 (fluoromethyl-(18F)-dimethyl-2-hydroxyethyl-ammonium) is presented as a solution for injection of F18-choline. It has a period of 109.8 minutes and emits positonic radiation of maximum energy 0.633 MeV, followed by annihilation photon radiation of 0.511 MeV.

8.2.2 Method of administration

The radionuclide will be administered intravenously.

8.2.3 Labelling

It will include the following information:

- • Name of preparation: F18-choline, solution for injection

- • Study reference: APACH2
- • Route of administration: IV
- • Batch number
- • Volume and activity administered
- • Name of Sponsor
- • Radioactivity pictogram

8.2.4 RPM Circuit for the Centre François Baclesse

- ✓ Reception: Fluorochol® will be delivered to the radiopharmacy airlock at the François Baclesse Center like any radioactive medication.
- ✓ Storage: Fluorochol®, for radiation protection and sterility purposes, will be handled in the radiopharmacy preparation room in a leaded UNIDOSE® distribution enclosure (Trasis).
- ✓ Preparation: Fluorochol® is a ready-to-use radiopharmaceutical specialty. Dispensing will be carried out in the radiopharmacy premises (class D controlled atmosphere zone, in accordance with Good Preparation Practices).
- ✓ Dispensing method: The nominative dispensing of the dose to be administered will be carried out using the UNIDOSE® dose distributor (Trasis). Dose traceability (batch number, prescription number, patient identification, dose dispensed, dose administered, expiry date, etc.) will be carried out on the VENUS® nuclear medicine software (Nicesoft).
- ✓ Accounting allowing an evaluation of compliance (accounting form): Taking into account the radioactive nature of Fluorochol® (physical period 110 minutes) and the single injection in the department, compliance will be evaluated thanks to the traceability carried out on the VENUS® nuclear medicine software (Nicesoft).

8.2.5 RPM Circuit for Brest University Hospital

- ✓ Reception: CISCholine® will be delivered to the nuclear medicine department of the Morvan hospital at the Brest University Hospital like any fluorinated radioactive drug.
- ✓ Storage: CISCholine®, for radioprotection and sterility purposes, will be handled within the radiopharmacy preparation room in a TEMA SINERGIE class A leaded enclosure equipped with a µDDSA® (TEMA SINERGIE) for dose distribution .
- ✓ Preparation: CISCholine® is a ready-to-use radiopharmaceutical specialty. Dispensing will be carried out in the radiopharmacy premises (class D controlled atmosphere zone, in accordance with Good Preparation Practices).
- ✓ Dispensing method: The nominative dispensing of the dose to be administered will be carried out using the µDDSA® dose distributor (TEMA SINERGIE). The traceability of the dose (batch number, prescription number, patient identification, dose dispensed, dose administered, expiry date, etc.) will be carried out on the nuclear medicine software GERA® (Theleme) initially then XPLORE (EDL) in a second step.
- ✓ Accounting allowing an evaluation of compliance (accounting form): Taking into account the radioactive nature of CISCholine® (physical period 110 minutes) and the single injection in the service, compliance will be evaluated thanks to traceability carried out on the nuclear medicine software GERA® (Theleme) firstly then XPLORE (EDL) secondly.

8.2.6 RPM Circuit for the Centre Eugène Marquis

- ✓ Reception: The CISCholine® will be delivered to the nuclear medicine department of the Eugène Marquis Centre
- ✓ Storage: For radiation protection and sterility purposes, the CISCholine® will be handled in the PET preparation room in a single-dose® (Trasis) leaded distribution chamber.
- ✓ Preparation: CISCholine® is a ready-to-use radiopharmaceutical specialty. The dispensing will be carried out in a single-dose® (Trasis) leaded distribution chamber.

- ✓ Method of dispensing: The nominal dispensing of the dose to be administered will be carried out using the single-dose dose® distributor (Trasis). The traceability of the dose (batch number, prescription number, patient identification, dose dispensed, dose administered, expiry date, etc.) will be carried out on the GERA® nuclear medicine software (Thelem).

In the event of a failure of the TRASIS dose distributor, the dose is dispensed manually in the radiopharmacy preparation room in a LemerPax high-energy Class A leaded chamber.

9 MEDICO-ECONOMIC STUDY

The medico-economic analysis will be carried out from the point of view of health insurance. From this perspective, only direct hospital costs will be included in the analysis. They will include the costs of diagnosis, the costs of surgery (type of surgical procedure, duration of intervention and hospitalization, anesthesia) and the cost of possible complications (hematomas, infections, damage to the recurrent laryngeal nerve, chronic hypoparathyroidism).

The efficacy endpoint will be the cure rate at one month (normalization of serum calcium at one month post-surgery). An incremental cost-effectiveness ratio will be estimated to establish the average cost needed to cure an additional person by moving from the usual strategy to the new strategy.

A sensitivity analysis of the uncertain parameters of the model will be performed to test the robustness of the cost-effectiveness ratio.

10 SAFETY

10.1 GENERAL RULES – INSTRUCTIONS

Safety management will be conducted according to the French regulatory requirements (Law No. 2012-300 of 05/03/2012 as amended by Ordinance No. 2016-800 of 16 June 20161)

10.2 DEFINITIONS

10.2.1 Adverse Event (AE)

Any untoward medical occurrence in a patient or clinical investigation subject administered a pharmaceutical product and which does not necessarily have a causal relationship with this treatment. An adverse event (AE) can therefore be any unfavorable and unintended sign (including an abnormal laboratory finding), symptom, or disease temporally associated with the use of a medicinal (investigational) product, whether or not considered related to it.

10.2.2 Adverse Reaction

All untoward medical occurrence in a patient or clinical investigation subject which participate to research involving the human person **related to the study procedure or to the experimental product.**

All untoward responses to an investigational medicinal product related to any dose administered.

10.2.3 SERIOUS Adverse event

A serious adverse event (SAE) is an AE that fulfils one or more of the following criteria:

- Results in death
- Is immediately life-threatening
- Requires in-patient hospitalization or prolongation of existing hospitalization
- Results in persistent or significant disability or incapacity

- Is a congenital abnormality or birth defect
- Is an important medical event*.

* Medical and scientific judgment should be exercised in deciding whether expedited reporting is appropriate in other situations, such as important medical events that may not be immediately life-threatening or result in death or hospitalization but may jeopardize the patient or may require intervention to prevent one of the other outcomes listed in the definition above. These should also usually be considered serious.

Some "hospitalization/prolonged hospitalization" are not considered to be reported as serious adverse event:

- Admission for administrative or social reasons
- Hospitalization predefined by the protocol
- Hospitalization for medical or surgical treatment planned before the beginning of the trial
- Passage to day hospital
- Hospitalizations for pre-existing signs and symptoms that have not been aggravated

These have not to be notified as SAE.

10.2.4 Suspected Unexpected Adverse Reaction

Any serious adverse reaction for which the nature, severity, frequency or outcome is not consistent with the applicable product information (e.g investigator's brochure for an unauthorized investigational product or summary of product characteristics for an authorised product).

Any serious adverse reaction for which the nature, severity, frequency or outcome is not consistent with the applicable product information, diagnostic or therapeutic study mandated procedure (non-invasive and/or invasive) evaluated in the protocol.

10.3 INVESTIGATOR'S RESPONSIBILITIES

10.3.1 Detection and registration of adverse events

All adverse events have to be searched, reported and recorded, processed and evaluated.

All adverse events occurring, once the informed consent form has been signed, **during treatment and 30 days after the last study drug administration,** must be recorded by the investigator.

The intensity of the events will be estimated according to the NCI-CTCAE version 5.0 classification (grade 1 to 5 toxicity). The intensity of adverse events not listed in this classification will be graded as follows:

- **Grade 1:** **mild** : Discomfort noticed but no disruption of normal daily activity
- **Grade 2:** **moderate** : Discomfort sufficient to reduce or affect daily activity; no treatment or medical intervention is indicated although this could improve the overall well-being or symptoms of the patient
- **Grade 3:** **severe** : Inability to work or perform normal daily activity; treatment or medical intervention is indicated in order to improve the overall well-being or symptoms; delaying the onset of treatment is not putting the survival of the patient at direct risk
- **Grade 4:** **Life-threatening/disabling :** An immediate threat to life or leading to a permanent mental or physical conditions that prevents work or performing normal daily activities; treatment or medical intervention is required in order to maintain survival
- **Grade 5:** **Death AE or resulting in death**

The investigator will specify for each event:

- ✓ The seriousness
- ✓ The date of start +/- the date of end of the event
- ✓ The duration
- ✓ The intensity
- ✓ The causality assessment
- ✓ The outcome

If a SAE occurs, the sponsor should be notified of the event awareness by the investigator without any delay. This will be done by mailing sponsor's Serious Adverse Event Report Form.

10.3.2 SAE reporting process

The investigator has to report the Sponsor of all serious adverse events occurring, once the informed consent form has been signed, **during treatment and 30 days after the last study drug administration**.

All late Serious Adverse Events and considered as reasonably related to the study treatment(s) or to the study procedure must be reported without delay limitation.

The investigator has to immediately report to the sponsor all serious adverse events with the exception of those that are identified as not requiring immediate reporting in the protocol or the investigator's brochure.

Pharmacovigilant contact : pv-cfb@baclesse.unicancer.fr
Tel : 02 31 45 40 78

This will be done by mailing sponsor's Serious Adverse Event Report Form, and it will be following by some report, as needed, containing all available information concerning the SAE.

The investigator will specify for each event:

- ✓ The description as clear as possible according to the medical terminology,
- ✓ The intensity,
- ✓ The date of start and the date of end of the event,
- ✓ Measures taken and the necessity or not for a corrective treatment,
- ✓ If the treatment of the study was discontinued or if dose was modified
- ✓ The outcome: For a non-fatal event, the event should be followed until resolution or return to the initial status or to stabilization of potential sequels,
- ✓ The **causality assessment**: he has to assign a causality of "related to study treatment", if there is a "reasonable possibility" that the study treatment caused the event, or "not related to study treatment" if there is "no reasonable possibility" that the study treatment caused the event. The investigator must also assess whether the serious adverse event is "possibly related" to any study mandated procedure or activity, or others concomitants treatments, or under study or any others disease.

The investigator should also, when possible, attach to the serious adverse event report:

- ✓ A copy of the hospital report/hospitalization prolongation,
- ✓ A copy of the autopsy report,
- ✓ A copy of the results of all additional exams performed, including relevant negative results together with the range of normal laboratory values,
- ✓ Any other document judged useful and pertinent (including imaging)

All these documents should be **anonymized**.
Additional information may be requested by the sponsor.

The investigator should document the event as best as possible, provide medical diagnosis where possible and establish a causal link between the serious adverse event and the research, the study treatment, the associated drugs, an underlying pathology, progression of the disease or other cause.
The investigator shall promptly provide the sponsor with additional information regarding serious adverse events as he becomes aware of them.
The investigator should follow the patient who has had a serious adverse event until resolution, stabilization at a level acceptable to the investigator or return to the previous status, even if the patient withdraws from study and inform the sponsor of the serious adverse event outcome.

10.3.3 Special cases

ADVERSE EVENT OF SPECIAL INTEREST

An adverse event of special interest (AESI) is a serious or non-serious adverse event that requires special attention and will be specifically searched. AESIs should be notified and followed as serious adverse events.

There is no AESI as part of this research.

REPORTING EXCEPTIONS

Serious adverse event not to be notified immediately

Any event that is part of the natural history of the disease (progression of the disease or hospitalization for progression of the disease) should not be notified to pharmacovigilance on the SAE form but has to be reported into the e-CRF.

Some "hospitalization/prolongation of hospitalization" are not considered as serious adverse events and do not require notification to pharmacovigilance (see last paragraph "Serious adverse event ").

PREGNANCY

If a woman starts a pregnancy as part of the study or in some cases if her partner participates in the study (drug that can reach the seminal line of the man), the investigator has to report pregnancy to the sponsor.

The investigator informs the sponsor who will send him an "pregnancy notification form”. This form must contain the expected date of delivery, the contact information of the obstetrician and the maternity ward provided for delivery if the pregnancy continues.

The investigator has to follow the patient until the end of the pregnancy or its interruption and notify the outcome to the sponsor. If the outcome of pregnancy falls within the definition of serious adverse events (spontaneous abortion with hospitalization, fetal death, congenital anomaly, ...) the investigator should follow the SAE reporting process.

If it is a paternal exposure, the investigator has to obtain the parturient consent to report pregnancy data.

11 STATISTICAL CONSIDERATIONS

11.1 SAMPLE SIZE

The primary endpoint is the proportion of patients for whom the first-line imaging technique (MIBI SPECT/CT or FCH PET/CT) allows guiding the surgical procedure appropriately, i.e. -say to decide on a true positive minimally invasive surgery and to obtain the patient's cure one month post-operatively.

Considering that the proportion of patients guided towards the right surgical strategy according to first-line imaging is 60% with MIBI SPECT/CT [Cheung, 2012] and 90% with FCH PET/CT [Quak , 2017], 50 evaluable patients (25 per arm) are necessary to detect such a difference in proportions with a risk of 5% and a power of 80% (one-sided test). It is planned to include 58 patients to anticipate 15% of possible non-evaluable patients. Based on the results of our published APACH-1 study [Quak, 2017] where a sensitivity of approximately 90% was observed with FCH PET/CT, we believe that a unilateral test is sufficient in the design of the design of APACH-2 to demonstrate the superiority of FCH PET/CT compared to MIBI SPECT/CT which has a sensitivity of approximately 60%.

11.2 STATISTICAL ANALYSIS

For the main objective, the proportions of patients oriented towards the right surgical strategy (decision for true positive minimally invasive surgery and obtaining the patient's cure) according to first-line imaging (MIBI SPECT/CT or FCH PET/CT) will be compared using a chi2 test with a one-sided alpha risk of 5%.

Qualitative variables will be described using numbers and percentages; Quantitative variables will be described using mean and standard deviation or quartiles as appropriate. Estimates will be provided with their bilateral 90% confidence intervals. We will calculate Cohen's kappa coefficients (with their 95% confidence interval) which will be considered strong if greater than 0.60, quasiperfect if greater than 0.80, perfect if equal to 1, and unsatisfactory otherwise.

The performance (sensitivity, specificity, error rate, positive and negative likelihood ratios) of the two interventions (FCH PET/CT and MIBI SPECT/CT) will be assessed using univariate analyses, Chi² test or Fisher's exact nonparametric test.

It is also planned to use multivariate logistic regression models to identify possible other parameters associated with the correct surgical strategy.

For patients who received both imaging techniques sequentially, we will describe their socio-demographic and clinical characteristics, as well as the proportion of minimally invasive surgeries and bilateral explorations. Depending on the number of patients, non-parametric comparisons will be made.

12 QUALITY CONTROL

In order to ensure the authenticity and credibility data in accordance to Good Clinical Practices (GCP), the sponsor will implement a quality assurance system that includes:

- the management of the test according to the procedures of the Clinical Research Unit
- the quality control of the data of the investigator site by the monitor whose role is to check the concordance and the coherence of the data of case report form of observation compared to the documents-source
- the provision if the funding provides for dedicated staff in the service to assist the investigator in the logistics of the study and the collection of data in the case report form.

13 ETHICS AND REGULATORY CONSIDERATIONS

The study will be conduct in accordance with the French Public Health Law, specially relating to research involving the biomedical human person of the Public Health Code, articles L1121-1 and following (Law No. 2012-300 of 05/03/2012 as amended by Ordinance No. 2016-800 of 16 June 2016), the Bioethics Law, the law related to the protection of physical persons for the treatments of personal data and related to information technology, database and liberties, the Helsinki declaration and the Good Clinical Practices

13.1 CLINICAL TRIAL AUTHORISATION

An authorization request will be sent by the sponsor to the French regulatory authorities before the study initiation:

- Ethics Committee (Committee for the Protection of Persons, CPP)
- Competent Health Authority (ANSM)

An Information will be given to the Competent Authority (ANSM), with transmission of the synopsis of the study and the favorable opinion of the CPP.

This study is under of the "Reference Methodology" (MR-001) in application of the provisions of article 54 paragraph 5 of the law of 6 January 1978 as amended relating to data processing, files and freedoms. This change was approved by decision of 5 January 2006.

The François Baclesse Center respects the regulations in force, in particular the rights of the persons being treated according to the EU regulation 2016/679 on the protection of data ("RGPD").

Any substantial modification in the protocol about objectives, design, population, evaluation, significant administrative modifications will need the coordinator approval, the sponsor approval, CPP approval and the competent authority authorization.

13.2 INFORMATION OF PATIENTS INVOLVED IN THE RESEARCH

Patients will be completely and faithfully informed with understandable words on the objectives and constraints of the research, potential risks, required measures for monitoring and safety, of their right to decline the participation in the study or the possibility to withdraw from the study at any time.

All these information are included in the informed consents form given to the patient: one for the main study, other for ancillary studies. The investigator, or the physician who represents him, will collect the signed written informed consent(s) before the definitive inclusion in the study. A copy of the information and consent form signed by the two parties will be given to the patient; the investigator will keep the second copy.

For any significant modification of the protocol related to the objectives of the research, its design, the population, the exams or significant administrative aspects, a new consent from each person participating to the research will be collected if needed.

13.3 INVESTIGATOR RESPONSIBILITIES

The Principal Investigator of each participating center is committed to conduct the clinical trial in accordance with the trial protocol and with the regulations in force, notably the decision of 24 November 2006 related to Good Clinical Practices.

The Principal Investigator is responsible for:

- ✓ Giving to Sponsor his/her curriculum vitae and that of co-investigators
- ✓ Identifying persons involved in the research in his/her team and defining their responsibilities
- ✓ Initiating the inclusion of patients after Sponsor authorization
- ✓ Making the maximum effort to include the required number of patients within the established recruitment period.

Each Investigator is responsible for:

- ✓ Obtaining the signed and dated informed consent and personally signing this consent for each participating patient before any procedure specific to the trial
- ✓ Regularly completing the Case Report Form (CRF) for each patient included in the trial and to allow to CRAs mandated by the Sponsor a direct access to source data in order to validate the data entered in the CRF
- ✓ Dating, correcting and signing any correction in the CRFs and data clarification forms (DCF)
- ✓ Accepting the regular monitoring visits of the monitor and eventual auditors mandated by the Sponsor or inspectors of supervisory authorities

Source documents, defined as any document or original item that allow to prove the existence or accuracy of a data or a fact recorded during the study, will be kept during 15 years by the investigator or the hospital if the source is a hospital medical record.

The archiving of the data will be the responsibility of the investigator and according to the legislation. The patient should keep the data and a patient identification list for a minimum of 15 years after the end of the study.

13.4 DATA CONFIDENTIALITY

The investigator will ensure the confidentiality of all information concerning the project for himself and for all persons involved in the conduct of the trial until the publication of the test results. This confidentiality obligation will not apply to information that the investigator will be required to provide to patients in the context of their participation in the trial or to information already published. The investigator will ensure not to publish, disclose or use, in any way, directly or indirectly, scientific or technical informations of the trial.

The study may not be the subject of any written or oral commentary without the agreement of the sponsor; all the information communicated or obtained during the realization of the test belonging in full right to the sponsor who can freely dispose of it.

14 DATA AND DOCUMENTS KEEPING

14.1 DATA ENTRY AND HANDLING

Data management will be performed by the Data Processing Center (CTD) of the North West Cancéropôle (Centre de Traitement des Données du Cancéropôle Nord-Ouest). The CTD provides a database management software dedicated to clinical research: Ennov Clinical (version 7.5.10, ENNOV / CLINSIGHT, 33155 Cenon, France).

This software package, which is based on an Oracle database architecture, is designed for the overall management of clinical and epidemiological studies, meets the regulatory requirements related to this type of study. The CTD Ennov Clinical instance is validated in its computing environment. A data validation plan will be developed jointly by the Clinical Research Unit and the Data Processing Center and will describe in detail the controls to be performed for each variable.

A database specific to the study will be created, tested and validated before the start of the study. All information required by the protocol must be recorded on the paper observation books - or on the electronic observation booklet - under the responsibility of the principal investigator and an explanation must be provided for each missing data item. The data will have to be entered in these notebooks as they are obtained, and the sponsor will take over the monitoring.

The data will then be checked by the CTD in accordance with the data validation plan. The database will be frozen after final quality control and then exported to the adequate format for statistical analysis according to an automated and validated procedure.

14.2 ARCHIVING

The sponsor must ensure the archiving of essential documents on the conduct of the study in conditions ensuring their safety, for the minimum duration provided by BPC, 15 years after the end of the research.

These documents are the protocol and annexes including any amendments, original signed information forms and consents, questionnaires, case report forms, follow-up documents, statistical analyzes, the final report of the study.

14.3 PUBLICATION POLICY

The results of this study, property of the Sponsor (Centre François Baclesse), will be published under scientific articles. Publications relating to or resulting from this research will be communicated and submitted for review by the study coordinators to all investigators.

The authors include investigators that have included most patients, the biostatistician who has performed the data analysis, the clinical researcher monitor and the participants who provided substantial contribution to the development of the study, the analysis and interpretation of results and / or the writing of the manuscript.

No publication or presentation of the results will be allowed without the agreement of all the parties. Each investigator will be author in the order determined by the number of eligible patients included. No publication or communication will be performed without the agreement of the coordinating investigator and the Sponsor with the obligation to mention the name of the Sponsor, the organism which financially supported the conduct of the trial.

Some dedicated publications for ancillary studies will be also performed.

This work will be the property of all authors and will be at their disposal for transversal communications and publications.

Publications related to the results of potential ancillary studies need prior approval of the coordinating investigator and methodologist and will be done after the publication of the main study, which should be cited as reference.

15 FUNDING AND INSURANCE

15.1 FUNDING

Any additional costs referred to the Code of Public Health are being negotiated between the CFB and the representative of the institution, taking into account the financial resources available to the CFB in the frame of its public promotion activities.

However, the CFB will ensure the study implementation and supply of the following material (protocol, CRF, investigator file) needed to the conduct of the study.

In the case of equipment or treatments are provided by other partners, the conditions must be specified in the study agreement.

15.2 INSURANCE

The sponsor has subscribed for the duration of the study an insurance covering his own liability and that of any physician involved in the realization of the study. It will also ensure full compensation for the harmful consequences to search for the person undergoing it and assigns, unless evidence against him that the damage is not attributable to its fault or that of any intervener, without that can be opposite the act of a third party or the voluntary withdrawal of the person who had originally agreed to participating to research (Article L 1121-10).

16 REFERENCES

Auquier P, Pernoud N, Bruder N, Simeoni MC, Auffray JP, Colavolpe C, François G, Gouin F, Manelli JC, Martin C, Sapin C, Blache JL. Development and validation of a perioperative satisfaction questionnaire. Anesthesiology. 2005 Jun;102(6):1116-23.

Beheshti M, Hehenwarter L, Paymani Z, Rendl G, Imamovic L, Rettenbacher R, Tsybrovskyy O, Langsteger W, Pirich C. 18F-Fluorocholine PET/CT in the assessment of primary hyperparathyroidism compared with 99mTc-MIBI or 99mTc-tetrofosmin SPECT/CT: a prospective dual-centre study in 100 patients. Eur J Nucl Med Mol Imaging. 2018 Sep;45(10):1762-1771.

Ciappuccini R, Morera J, Pascal P, Rame JP, Heutte N, Aide N, Babin E, Reznik Y, Bardet S. Dual-phase 99mTc sestamibi scintigraphy with neck and thorax SPECT/CT in primary hyperparathyroidism: a single-institution experience. Clin Nucl Med. 2012 Mar;37(3):223-8.

Giussani A, Janzen T, Uusijarvi-Lizana H, et al. A compartmental model for biokinetics and dosimetry of 18F-choline in prostate cancer patients. J Nucl Med 2012;53:985-993.

Hocevar M, Lezaic L, Rep S, Zaletel K, Kocjan T, Sever MJ, Zgajnar J, Peric B. Focused parathyroidectomy without intraoperative parathormone testing is safe after pre-operative localization with 18F-Fluorocholine PET/CT. Eur J Surg Oncol. 2017 Jan;43(1):133-137.

ICRP. Radiation dose to patients from radiopharmaceuticals. A fourth addendum to ICRP publication 53. 2013

Michaud L, Burgess A, Huchet V, Lefèvre M, Tassart M, Ohnona J, Kerrou K, Balogova S, Talbot JN, Périé S. Is 18F-fluorocholine-positron emission tomography/computerized tomography a new imaging tool for detecting hyperfunctioning parathyroid glands in primary or secondary hyperparathyroidism? J Clin Endocrinol Metab. 2014 Dec;99(12):4531-6.

Prabhu M, Kumari G, Damle NA, Arora G, Kumar P, Kumar R, Tripathi M, Bal C, Khadgawat R, Kumar C, Agarwal S. Assessment of the role of early dynamic PET/CT with 18F-fluorocholine in detection of parathyroid lesions in patients with primary hyperparathyroidism. Nucl Med Commun. 2018 Dec;39(12):1190-1196.

Quak E, Blanchard D, Houdu B, Le Roux Y, Ciappuccini R, Lireux B, de Raucourt D, Grellard JM, Licaj I, Bardet S, Reznik Y, Clarisse B, Aide N. F18-choline PET/CT guided surgery in primary hyperparathyroidism when ultrasound and MIBI SPECT/CT are negative or inconclusive: the APACH1 study. Eur J Nucl Med Mol Imaging. 2018 Apr;45(4):658-666.

Quak E, Lheureux S, Reznik Y, Bardet S, Aide N. F18-choline, a novel PET tracer for parathyroid adenoma? J Clin Endocrinol Metab. 2013 Aug;98(8):3111-2.

Rep S, Lezaic L, Kocjan T, Pfeifer M, Sever MJ, Simoncic U, Tomse P, Hocevar M. Optimal scan time for evaluation of parathyroid adenoma with [(18)F]-fluorocholine PET/CT. Radiol Oncol. 2015 Nov 27;49(4):327-33.

Rodger M, Ramsay T, Fergusson D. Diagnostic randomized controlled trials: the final frontier. Trials. 2012 Aug 16;13:137.

Thanseer N, Bhadada SK, Sood A, Mittal BR, Behera A, Gorla AKR, Kalathoorakathu RR, Singh P, Dahiya D, Saikia UN, Rao SD. Comparative Effectiveness of Ultrasonography, 99mTc-Sestamibi, and 18F-Fluorocholine PET/CT in Detecting Parathyroid Adenomas in Patients With Primary Hyperparathyroidism. Clin Nucl Med. 2017 Dec;42(12):e491-e497.

Treglia G, Piccardo A, Imperiale A, Strobel K, Kaufmann PA, Prior JO, Giovanella L. Diagnostic performance of choline PET for detection of hyperfunctioning parathyroid glands in hyperparathyroidism: a systematic review and meta-analysis. Eur J Nucl Med Mol Imaging. 2019 Mar;46(3):751-765.

Udelsman R, Lin Z, Donovan P. The superiority of minimally invasive parathyroidectomy based on 1650 consecutive patients with primary hyperparathyroidism. Ann Surg. 2011 Mar;253(3):585-91.
