# Supplementary material for: F18-Choline PET/CT or MIBI SPECT/CT in the Surgical Management of Primary Hyperparathyroidism: A Diagnostic Randomized Clinical Trial
Source: JAMA Otolaryngol Head Neck Surg. 2024 Jun 20;150(8):658–65. doi: 10.1001/jamaoto.2024.1421 (PMC11190825; doi:10.1001/jamaoto.2024.1421)
Supplement: Supplement 2. — eMethods. Imaging Procedures [file jamaotolaryngolheadnecksurg-e241421-s002.pdf]

Supplemental Online Content

Quak E, Lasne-Cardon A, Cavarec M, et al. F18-Choline PET/CT or MIBI SPECT/CT in the surgical management of primary hyperparathyroidism: a diagnostic randomized clinical trial. *JAMA Oncol*. Published online June 20, 2024. doi:10.1001/jamaoto.2024.1421

eMethods. Imaging Procedures

This supplemental material has been provided by the authors to give readers additional information about their work.

© 2024 Quak E et al. *JAMA Oncol.*

eMethods. Imaging Procedures

FCH PET/CT procedure

Patients fasted for 4 hours. Sixty minutes after intravenous injection of 1.5 MBq/kg of FCH, a low-dose CT was performed covering the neck and upper chest, followed by the PET acquisition. The injected activity and the exact delay between injection and the start of acquisition were recorded. The PET/CT acquisition was performed on a Vereos digital PET/CT system (Philips Healthcare), 2 bed positions of 10 minutes in 3D list-mode, or a Biograph mCT Flow system (Siemens Healthineers), 10 minutes continuous acquisition of the neck and upper chest. Before the start of the study, the acquisition protocols of the different PET systems used were harmonized in order to obtain similar results on each system. Image analysis was performed on dedicated Syngo.via workstations (Siemens Healthineers) in a double-blinded fashion. Care was taken that the first reader did not leave any tags on the images (for example screen shots, arrows, size or SUV measurements) that could guide the second reader.

MIBI SPECT/CT procedure

MIBI SPECT/CT was performed on Siemens SPECT/CT systems (Siemens Healthineers) in all centres. All centers used the dual phase protocol: after intravenous injection of 740 MBq of MIBI, an early pinhole acquisition of the anterior lower neck was performed 10 minutes post-injection, followed by a late SPECT/CT acquisition of the neck and upper chest 90 minutes post-injection. Image analysis was performed on dedicated Syngo.via workstations (Siemens Healthineers) in a double-blinded fashion.
