# Supplementary material for: F18-Choline PET/CT or MIBI SPECT/CT in the Surgical Management of Primary Hyperparathyroidism: A Diagnostic Randomized Clinical Trial
Source: JAMA Otolaryngol Head Neck Surg. 2024 Jun 20;150(8):658–65. doi: 10.1001/jamaoto.2024.1421 (PMC11190825; doi:10.1001/jamaoto.2024.1421)
Supplement: Supplement 3. — Data Sharing Statement [file jamaotolaryngolheadnecksurg-e241421-s003.pdf]

Data Sharing Statement

Data
Data available: Yes
Data types: Deidentified participant data
How to access data: e.quak@baclesse.unicancer.fr
When available: With publication

Supporting Documents
Document types: None

Additional Information
Who can access the data: Researchers with an approved study protocol
Types of analyses: meta-analysis
Mechanisms of data availability: after approval of a proposal, or with a signed data access agreement
